# Visualizing the Heterogeneity in Homogeneous Supramolecular Polymers

**DOI:** 10.1021/jacs.4c03562

**Published:** 2024-07-10

**Authors:** Emmanouil Archontakis, Shikha Dhiman, Miao Zhang, Marle E. J. Vleugels, E. W. Meijer, Anja R. A. Palmans, Peter Zijlstra, Lorenzo Albertazzi

**Affiliations:** † Department of Biomedical Engineering, and Institute for Complex Molecular Systems, Eindhoven University of Technology, 5600MB Eindhoven, The Netherlands; ‡ Laboratory of Macromolecular and Organic Chemistry, and Institute for Complex Molecular Systems, Eindhoven University of Technology, P.O. Box 513, 5600 MB Eindhoven, The Netherlands; § School of Chemistry and RNA Institute, The University of New South Wales, Sydney, New South Wales 2052, Australia; ∥ Department of Applied Physics and Science Education, and Institute for Complex Molecular Systems, Eindhoven University of Technology, 5600MB Eindhoven, The Netherlands

## Abstract

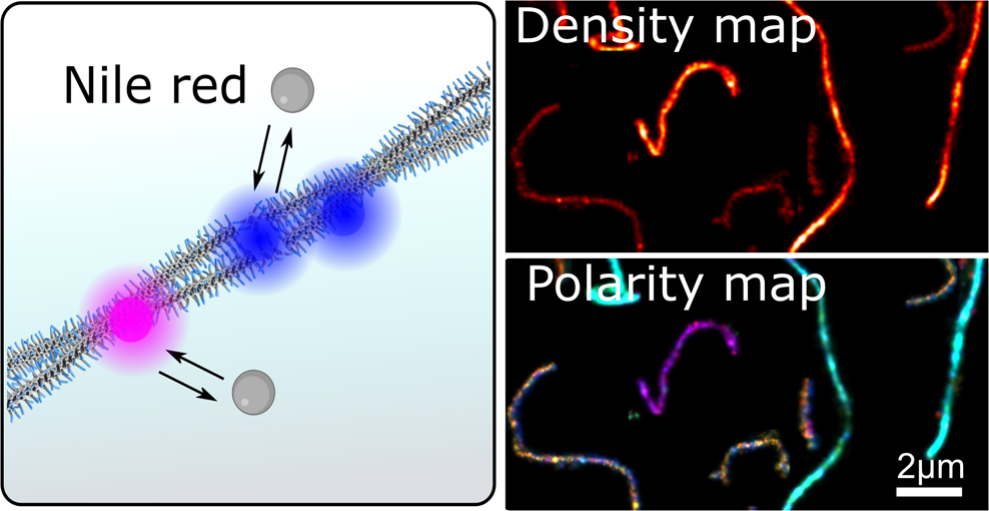

The dynamic properties of supramolecular polymers enable new functionality beyond the limitations of conventional polymers. The mechanism of the monomer exchange between different supramolecular polymers is proposed to be closely associated with local disordered domains within the supramolecular polymers. However, a direct detection of such heterogeneity has never been experimentally probed. Here, we present the direct visualization of the local disordered domains in the backbone of supramolecular polymers by a super-resolution microscopy technique: Nile Red-based spectrally resolved point accumulation for imaging in nanoscale topography (NR-sPAINT). We investigate the local disordered domains in trisamide-based supramolecular polymers comprising a (co)assembly of benzene-1,3,5-tricarboxamide (BTA) and a variant with one of the amide bonds inverted (iBTA). The NR-sPAINT allows us to simultaneously map the spatial distribution and polarity of the local disordered domains along the polymers with a spatial precision down to ∼20 nm. Quantitative autocorrelation and cross-correlation analysis show subtle differences in the spatial distribution of the disordered domains between polymers composed of different variants of BTA monomers. Further, statistical analysis unraveled high heterogeneity in monomer packing at both intra- and interpolymer levels. The results reported here demonstrate the necessity of investigating the structures in soft materials at nanoscale to fully understand their intricacy.

## Introduction

Supramolecular polymers represent a unique class of synthetic polymers composed of self-assembled monomers via reversible and highly directional noncovalent interactions, such as hydrogen bonds.^1,2^ The strikingly dynamic properties of these materials arise from the reversible bonds and open up the prospect of many new applications beyond the limitation of conventional polymers, such as self-healing materials and tissue engineering.^3^ Therefore, there is a growing interest in understanding supramolecular polymer dynamics and in controlling their formation. In a simple way, supramolecular polymers can be divided into two categories. The random coil polymers are typically formed by an isodesmic mechanism, and their dynamics is rather simple, while the highly ordered supramolecular polymers are formed by a cooperative nucleation–elongation mechanism.^1,4^ These highly ordered polymers are in many aspects like natural fibers, and their dynamics as well as the homogeneity in structure are the subject of many studies. Once the fibers are formed, dynamic properties arise from the continuous monomer exchange between the fibers. Many mechanisms of monomer exchange are possible; however, this is difficult to prove experimentally for many materials. The two main hypotheses presented in the literature for these fibers involve either the growth and shrinkage of the polymer from the polymer ends or the fragmentation and recombination of large blocks of the fiber.^5^ Recently, one unexpected mechanism of dynamics was unraveled on supramolecular polymers made of water-soluble benzene-1,3,5-tricarboxamides (BTAs). In this case, the BTA monomers can exchange homogeneously throughout the backbones of the polymer by single-monomer insertion.^6^ Computational studies suggest that the monomers exit the polymer backbone only at the disordered domains within the polymer, indicating that areas of disorder or defects in the polymer structure are drivers of dynamics.^7,8^ The theoretical models of supramolecular polymers based on Seargent and soldier experiments show that ordered domains have a characteristic length, also indicated as cooperativity length, after which order is lost and more disorder domains are present.^9−11^

This new paradigm, based on the presence of defects along the polymer structure, changed the view on supramolecular fiber dynamics, and it is now applied beyond BTAs, both in the materials and biology realm.^5^ Even for microtubules, long thought to be static and highly regular, increasing evidence is presented that they are much more labile and that their structures are corrected through defects.^12^ In other words, microtubules as well as many other biological fibers are less homogeneous than was proposed.

However, the existence of such disordered domains in synthetic supramolecular polymers has never been visualized. The challenge lies in the characterization of the structure of such soft materials at the nanoscale without perturbing their organization. Techniques such as transmission electron microscopy (TEM), cryogenic transmission electron microscopy (cryo-TEM), or atomic force microscopy (AFM) allow unveiling fine details of material structure with extraordinary resolution.^13−15^ However, there are several limitations related to their perturbation of the sample (e.g., freezing or drying) and their lack of molecular specificity. Alternatively, fluorescence microscopy has been used to image polymers labeled by fluorophores, having the unique advantages of chemical specificity enabled by versatile labeling, noninvasiveness, dynamics probing, and compatibility to correlative imaging with other microscopic techniques.^16,17^ Yet, the optical approach has long been limited by diffraction-limited resolution. Recently, with the advancing of super-resolution microscopic (SRM) techniques, unprecedented structural details and dynamics of the supramolecular polymers have been unraveled by, e.g., stochastic optical reconstruction microscopy (STORM) and structured illumination microscopy (SIM).^18−21^

Most super-resolution microscopic techniques require labeling of the monomers, potentially affecting their assembly behavior. However, point accumulation for imaging in nanoscale topography (PAINT) does not require prelabeling of the molecules but rather relies on transient binding between fluorescent probes and the target molecules.^22,23^ Transient binding of the fluorescent probe allows one to sequentially localize binding events to the target molecule with high precision, such that the accumulation of many locations forms a map of the target structures with a spatial resolution of 5–20 nm, well beyond the optical diffraction limit.^24^ Various probes with relatively low affinity, allowing transient binding specifically to various target molecules, have been developed to extend the applications of the PAINT methods in imaging cells, soft materials, and solid-state materials.^25−28^ Recently, the multiplexing capacity of PAINT has improved tremendously by resolving the spectrum of each single-molecule event.^29^ This is achieved by adding a transmission grating in the detection path, so that spatial and spectral information on each binding events are extracted from the zeroth- and first-order diffraction.^30^

**Figure 1:**
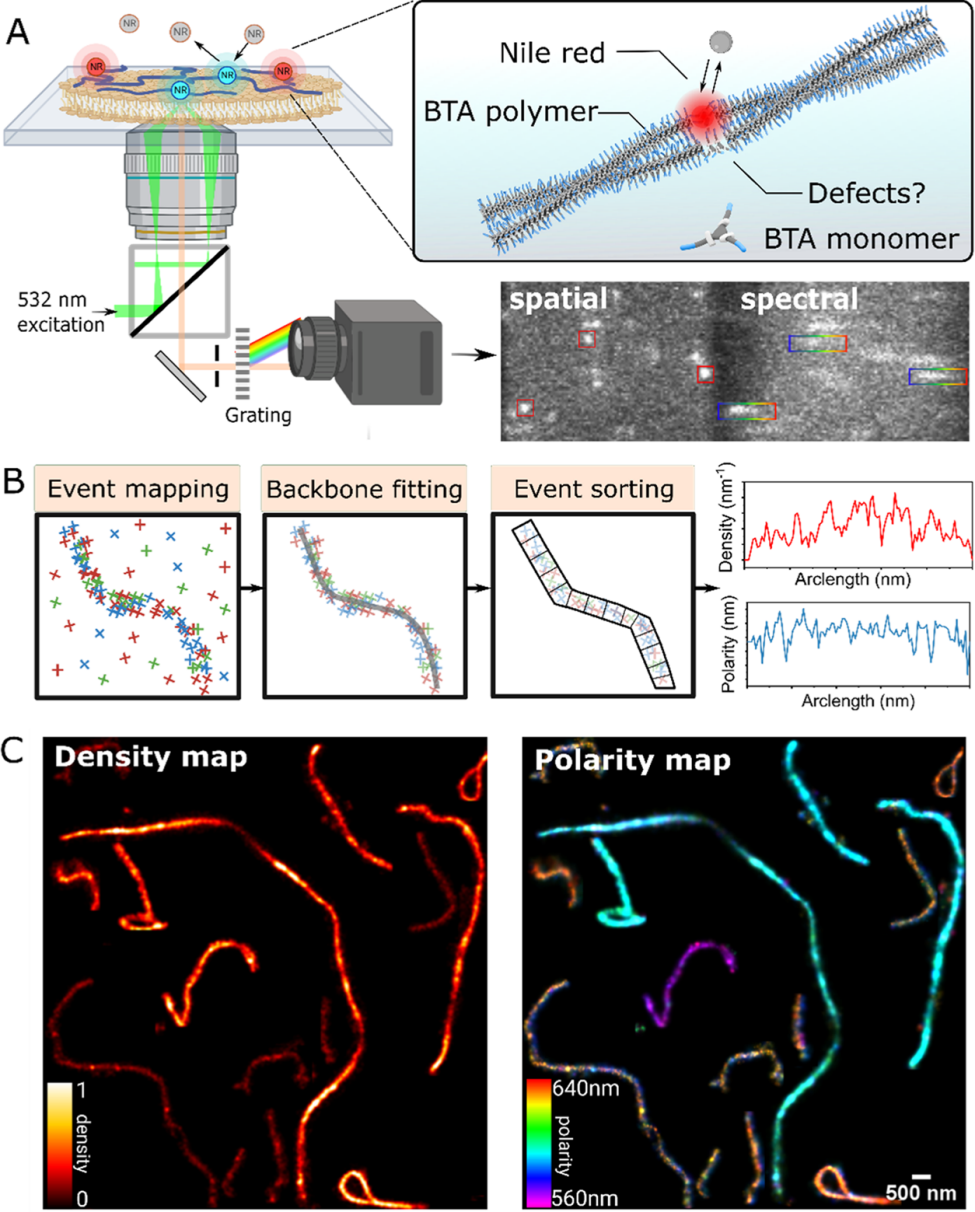
Nile Red-spectrally resolved PAINT method for mapping the structure of supramolecular polymers. (A) Nile Red excitation takes place using a TIRF setup. Each transient Nile Red binding on immobilized fibers generates a fluorescent event signal that is directed on a diffraction optical element (grating). The spatial and spectral information on the binding event is then obtained simultaneously from the zeroth-order and first-order diffractions. (B) After background removal and fiber identification by a clustering algorithm, the fiber backbone is identified, and binding events are sorted into 24 nm sections. This allows us to extract the event density and the polarity profile of each polymer fiber and perform further quantitative analysis. (C) Typical density map and polarity map of the polymers of iBTA.

In this work, we used PAINT to visualize heterogeneities along the backbone of individual BTA-based fibers to solve the long-standing issue of supramolecular structural defects. By using a solvatochromic probe, Nile Red (NR), and spectral detection, we were able to visualize the polarity and accessibility of the BTA structure at the nanoscale without the need for covalent labeling. NR is a hydrophobic dye that preferably binds to the hydrophobic cores of supramolecular polymers and fluoresces strongly in hydrophobic environments.^31,32^ Its emission wavelength shifts depending on the polarity of the environment and is thus widely used as a lipid probe.^33,34^ Using spectrally resolved PAINT with free diffusing NR probes, mapping of the hydrophobic landscape of materials has been demonstrated to be feasible.^35^ Here, we probe for the first time, using Nile Red-spectrally resolved PAINT (NR-sPAINT), the inhomogeneities of the disordered structure and polarity of the supramolecular polymer as illustrated in Figure 1. In contrast to previous SRM studies on supramolecular polymers, the free NR dyes here can fit into any of the hydrophobic pockets present in supramolecular polymers and thereby reveal nanoscale differences in the structure. Using this method, we unravel the local hotspots for NR binding along the polymer. The spatial distribution of the local hotspots varies among supramolecular polymers composed of different variants of BTA monomers. Further quantitative analysis of the spatial and polarity properties along the polymers unraveled a high heterogeneity at both interfiber and intrafiber levels with unprecedented details.

## Results and Discussion

### BTA Assembly and Imaging

**Figure 2:**
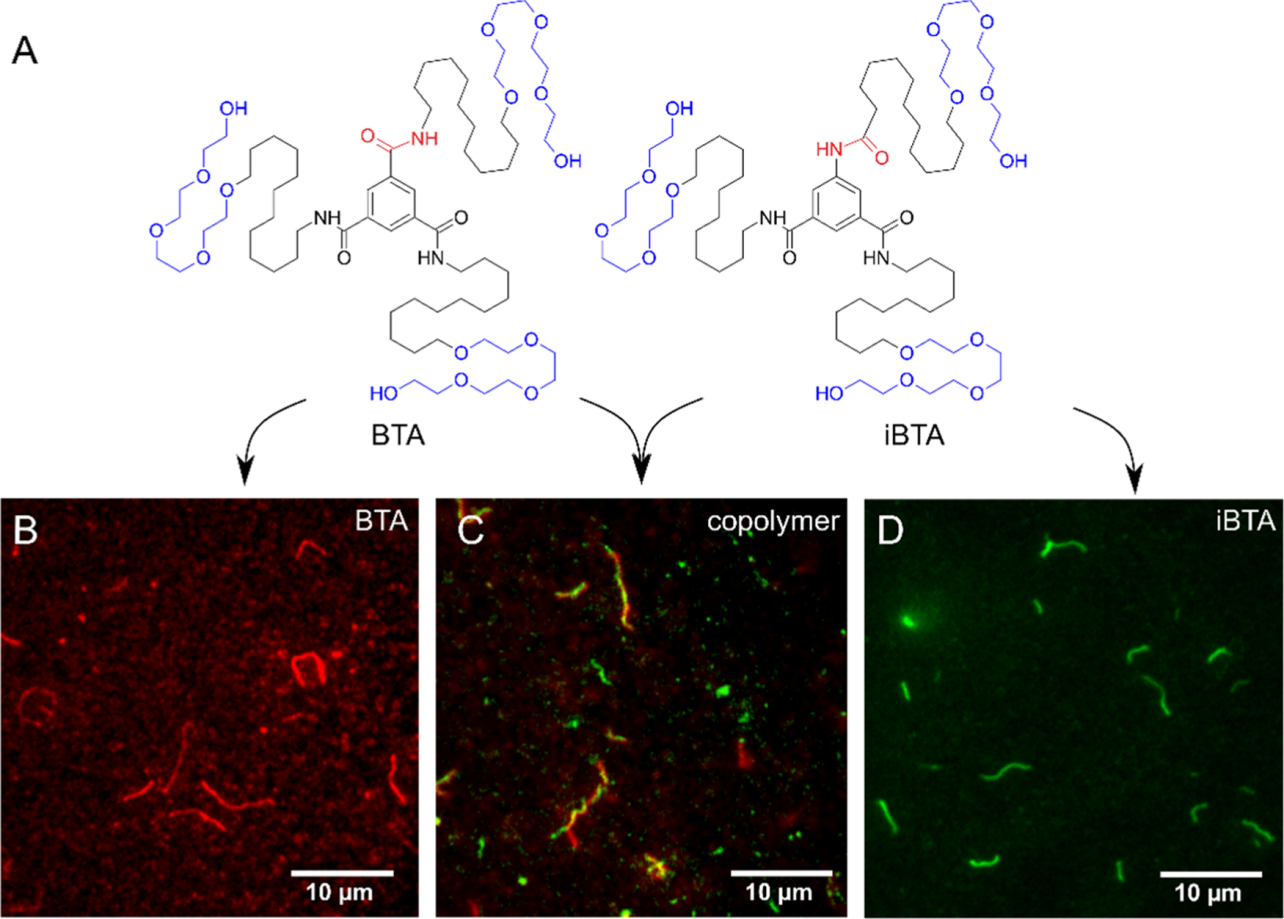
Structure and fluorescence images of supramolecular polymers. (A) Chemical structures of monomers iBTA and BTA. (B–D) TIRF images of BTA homopolymer (B), BTA–iBTA copolymer (C), and iBTA homopolymer (D). 2.5 μM, 1× PBS with 5% BTACy5 and/or iBTACy3.

Supramolecular polymers based on benzene-1,3,5-tricarboxamide (BTA) have been extensively studied due to their fascinating one-dimensional (1D) architecture, tunable dynamics, and various functional applications in aqueous environment.^4,36−39^ In this study, we used BTA and a variant, in which the connectivity of one amide to the central benzene core is inverted, inverted amide BTA (iBTA), as model systems to probe the distribution of disordered domain along the polymer chain. The two molecules have identical molecular weights and almost identical chemical structures as shown in Figure 2A.^40^ Both BTA and iBTA monomers assemble into 1D fibers due to hydrophobic interactions, with amide groups forming hydrogen bonds to control the directionality of the assembly process. The outer hydrophilic ethylene glycol (PEG) chains solubilize the structure in water. The only difference between BTA and iBTA is that one of the amide groups (red in Figure 2A) is inverted in iBTA molecules, which may affect hydrogen bond formation as the *C*_3_ symmetry is broken.

To observe the self-assembly of the monomers, we labeled BTA and iBTA monomers with Cy5 and Cy3 dyes, respectively, to perform fluorescence imaging of the BTA, iBTA homopolymers, and BTA/iBTA (1:1) copolymers by total internal reflection fluorescence (TIRF) microscopy. As shown in Figure 2B–D, all three types of monomers polymerized into fibers in water. Note that the distribution of BTA and iBTA is not consistent throughout a single copolymer fiber (Figure 2C). This is due to a reduced solubility of iBTA, which was reported in our previous study of BTA–iBTA copolymer using ensemble characterization methods.^40^ However, the TIRF measurement cannot resolve the polymer assembly at nanoscale. In addition, hydrogen/deuterium exchange followed by mass spectrometry (HDX-MS)^41,42^ was used to investigate monomer dynamicity within the supramolecular polymers (Figure S1 and Table S1). Here, a similar overall kinetics of exchange was observed for the polymers. Whereas BTAs are known to form double helical fibers,^43^ the exact morphology of iBTA fibers has not been elucidated. In addition, the inversion of one amide may affect the ability to form hydrogen bonds. How these subtle changes then affect coassemblies of BTA and iBTA remains unknown, because such subtle differences cannot be precisely detected at the molecular level by ensemble methods. This motivated us to switch to super-resolution single-molecule imaging to distinguish the elusive differences between these three materials.

### BTA Super-Resolution Imaging

To visualize the differences in assembly of the BTA, iBTA homo-, and copolymers at a single molecular level, we attach the polymer fibers to the glass coverslip using a supported lipid bilayer (SLB) through the streptavidin–biotin interaction. To address the potential local distortions in supramolecular polymers induced by biotin–streptavidin anchoring points, we mitigate this by reducing the biotin content in SLB to 0.1% DOPE-Bio and in supramolecular polymers to 1% BTABio. This concentration ensures minimal anchoring points, sufficient for attaching fibers to the surface while keeping them in solution, thus preserving their dynamical structural characteristics and minimizing any potential distortion. Details are given in the Materials and Methods section. After adding the NR dye in solution, the samples are imaged on an inverted microscope as depicted in Figure 1A. The hydrophobic interaction between NR and the supramolecular polymers in water results in transient binding and fluorescent emission. The emission fluorescence from NR is collected by a high numerical aperture objective lens and split by a transmission grating into zeroth-order and first-order diffractions, which allows us to capture the spatial locations and corresponding spectra of the binding events simultaneously. As can be seen in Figure S2, NR favors its binding to polymers over SLB, which allows us to eliminate background events by density filters in postimage processing. For each sample, 32,000 frames were taken with an exposure time of 40 ms to ensure good coverage of binding events in the polymers. Processing of the image stacks by standard single-molecule localization algorithms with Fiji plugins, Thunderstorm, and RainbowStorm,^44,45^ we obtain a matrix of event data with spatial coordinates, emission photons, spectral wavelength, etc., to allow us to reconstruct the super-resolved images by summing all events (Figure S2). Then, to perform quantitative analysis at a single-fiber level, postprocessing of the data was performed using a previously reported workflow.^6^ In brief, the procedure involves three steps as depicted in Figure 1B: (i) clustering algorithms to remove nonspecific background detections and identify fibers; (ii) backbone fitting to extract the arc length and curvature of the fibers; and (iii) sorting events into 24 nm sections to generate event density and polarity maps of each polymer fibers. Figure 1C shows typical reconstructions of the event density and polarity maps of the iBTA polymers. We could already see the heterogeneity in both event density and polarity map at inter- and intrafiber levels. To better access this heterogeneity, we performed quantitative analysis of the event density and polarity of fibers.

### Heterogeneity in Density Maps of Polymer Fibers

**Figure 3:**
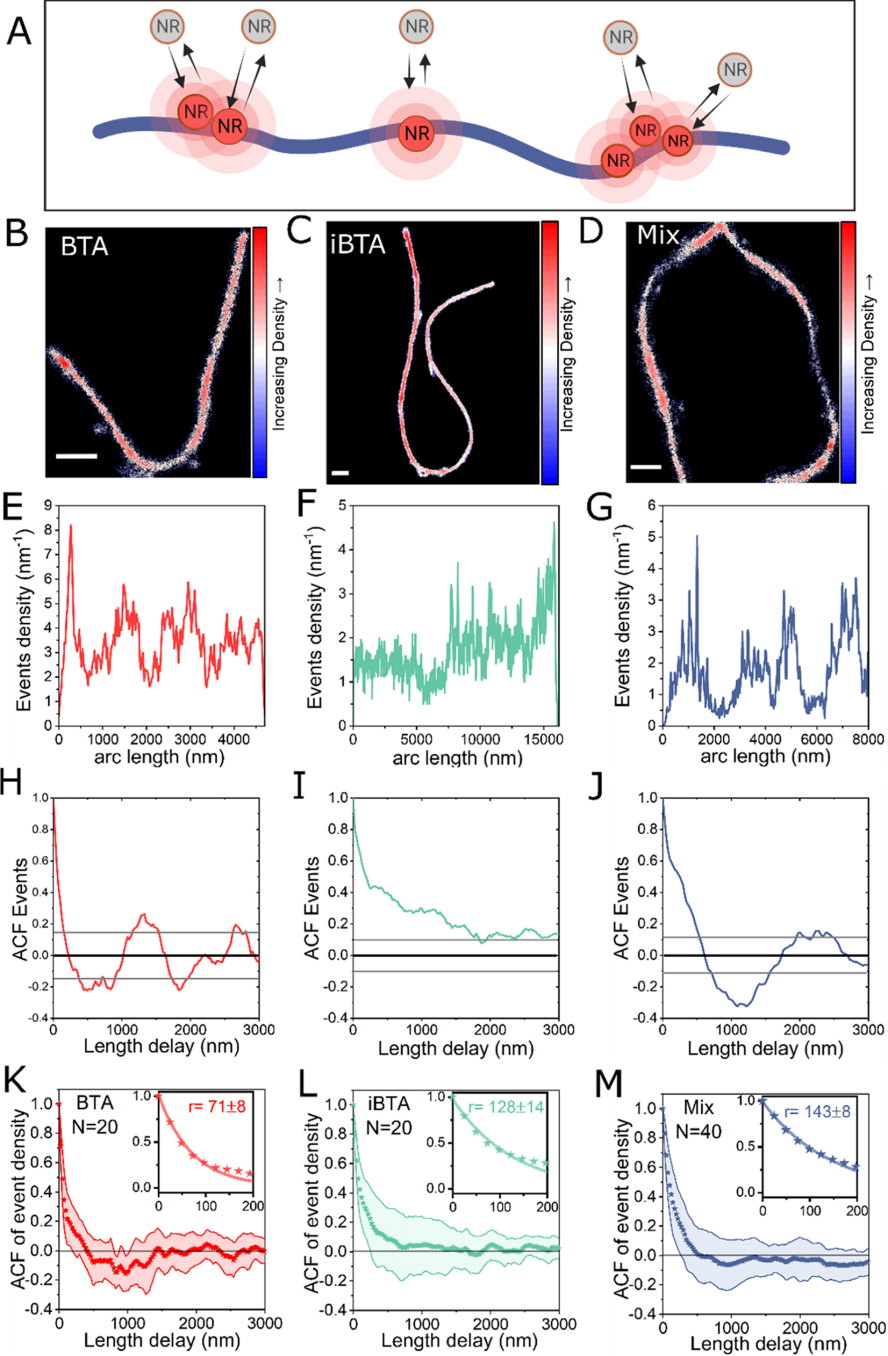
Heterogeneity of event density along the polymer fibers, between fibers, and between polymer categories. (A) Schematic representation of NR dyes probing the hydrophobic pockets in the polymer fiber. (B–D) Typical density maps of BTA, iBTA, and BTA–iBTA copolymers. (E–G) Event density profiles of the polymer fibers shown in panels (B–D). (H–J) Autocorrelation functions (ACFs) of the event density profiles shown in panels (E–G). Gray lines are the confidence bounds. (K–M) Averaged autocorrelation functions of the event density profiles of BTA, iBTA, and mixed polymers of 20, 20, and 40 polymer fibers, respectively. Insets in panels (K–M) show zoomed-in images of ACF curves with single-exponential decay fitting. The characteristic decay lengths for BTA, iBTA, and BTA–iBTA copolymers are 71 ± 8, 128 ± 14, and 143 ± 8 nm, respectively. The scale bar in panels (B–D) is 500 nm.

First, we examined the distribution of hydrophobic pockets in the polymer fibers. As shown in Figure 3A, NR binding events were mapped along the polymer to visualize the hydrophobic pockets. Typical event density maps of BTA, iBTA, and BTA–iBTA copolymer are shown in Figure 3B–D. Examples of other density maps of polymers can be found in Figures S4–S6. A higher density of events indicates a higher accessibility of the pocket for hydrophobic NR dyes. A similar observation was made in our previous work in the single-chain polymeric nanoparticles.^46^ We attribute such hotspots to local disordered regions of monomer stacking, where the hydrophobic core of the polymer is more exposed, leading to a higher binding frequency of NR dyes. Fluctuations in event density are clearly visible in all 3 cases of polymers, indicating that local defective regions along the polymer are generic to this type of supramolecular polymer. To get a quantitative insight into the spatial distribution of the defective regions, event density profiles of the 3 different polymers are plotted in Figure 3E–G, where we can see that fluctuations of event density along the fibers vary in pattern. The event rate along the pictured BTA polymer and the BTA–iBTA copolymer fluctuates in a patchy pattern as shown in Figure 3E,G, whereas on the pictured iBTA polymer, a staircase-like pattern is observed as shown in Figure 3F. We further perform autocorrelation function (ACF) analysis of the event density profiles to extract characteristic decay lengths and periodicities among many polymers from the same type, as shown in Figure 3H–J. Generally, a higher ACF value indicates a stronger correlation in event density and/or polarity across a certain arc length. Among the 3 selected polymers, the ACF of BTA polymer decays on the shortest length scale (Figure 3H). The ACF further shows a weak oscillatory pattern, which might indicate periodicity in the patchy structure. In stark contrast, for the iBTA polymer, ACF values decay much slower with no evidence of periodic peaks (Figure 3I). Such ACF curve reflects a slowly varying density of hydrophobic sites on longer length scales. The ACF of the BTA–iBTA copolymer has an overall shape similar to the BTA polymer but with a longer decay length and periodicity (Figure 3J). To get a statistical overview of the polymers, we perform the ACF analysis on multiple polymers in each category (*n* = 20 for BTA, *n* = 20 for iBTA, and *n* = 40 for BTA–iBTA). The advantage of the ACF analysis is that it can be easily averaged, allowing us to find statistically relevant features among many samples. As shown in Figure 3K–M, the averaged ACFs of all types of polymers decay to zero with characteristic decay lengths of 71 ± 8 nm for BTA, 128 ± 14 nm for iBTA, and 143 ± 8 nm for the BTA–iBTA copolymer. We also extracted the decay length on individual fibers. A statistically significant difference was only found between BTA and copolymer due to the large heterogeneity among fibers (Figure S7). The mean values of ACF for BTA decay quickly to negative values (anticorrelation), implying that BTA polymers are more likely to have patchy structures with relatively short-length correlation. For iBTA polymers, the mean values of ACF decay geometrically slower to zero, which implies that the iBTA polymers are more likely to have structures with long-range correlations. The BTA–iBTA copolymers have a resemblance to both BTA and iBTA with the longest-length correlation. However, it is important to note that the large standard deviations accompanying the ACF curves (shaded area in Figure 3K–M) imply a similar but high degree of heterogeneity among all types of polymers. This highlights the importance of characterization at the single-fiber level.

We attribute the subtle differences in the spatial distribution of the disordered domains between BTA and iBTA polymers to differences in monomer stacking mechanism and morphology. We speculate that the iBTA monomers are highly flexible in their structure with three long arms capable of various conformational states. The major contribution of long-range order in the supramolecular polymerization of iBTA arises from π–π stacking of benzene cores and hydrogen bonding between amide groups. Since the triamides in iBTA are not equivalent, differential packing arrangements can arise.^47^ In addition, the recently discovered double helical structure of BTA polymers in water^43^ indicates that the hydrogen bond packing is far from the regular 3-fold helical hydrogen bonds observed in crystal structures. Mismatches of monomers are also expected in the BTA system. Whether BTA or iBTA polymers have more mismatches? Observations made by our method show that the event densities are almost identical among all 3 types of polymers (Figure S7), implying a similar level of mismatches among the polymers. However, the microscopic view of the distributions of the disordered domains shows subtle differences between BTA, iBTA, and copolymers as discussed above.

### Heterogeneity in Polarity Maps of Polymer Fibers

**Figure 4:**
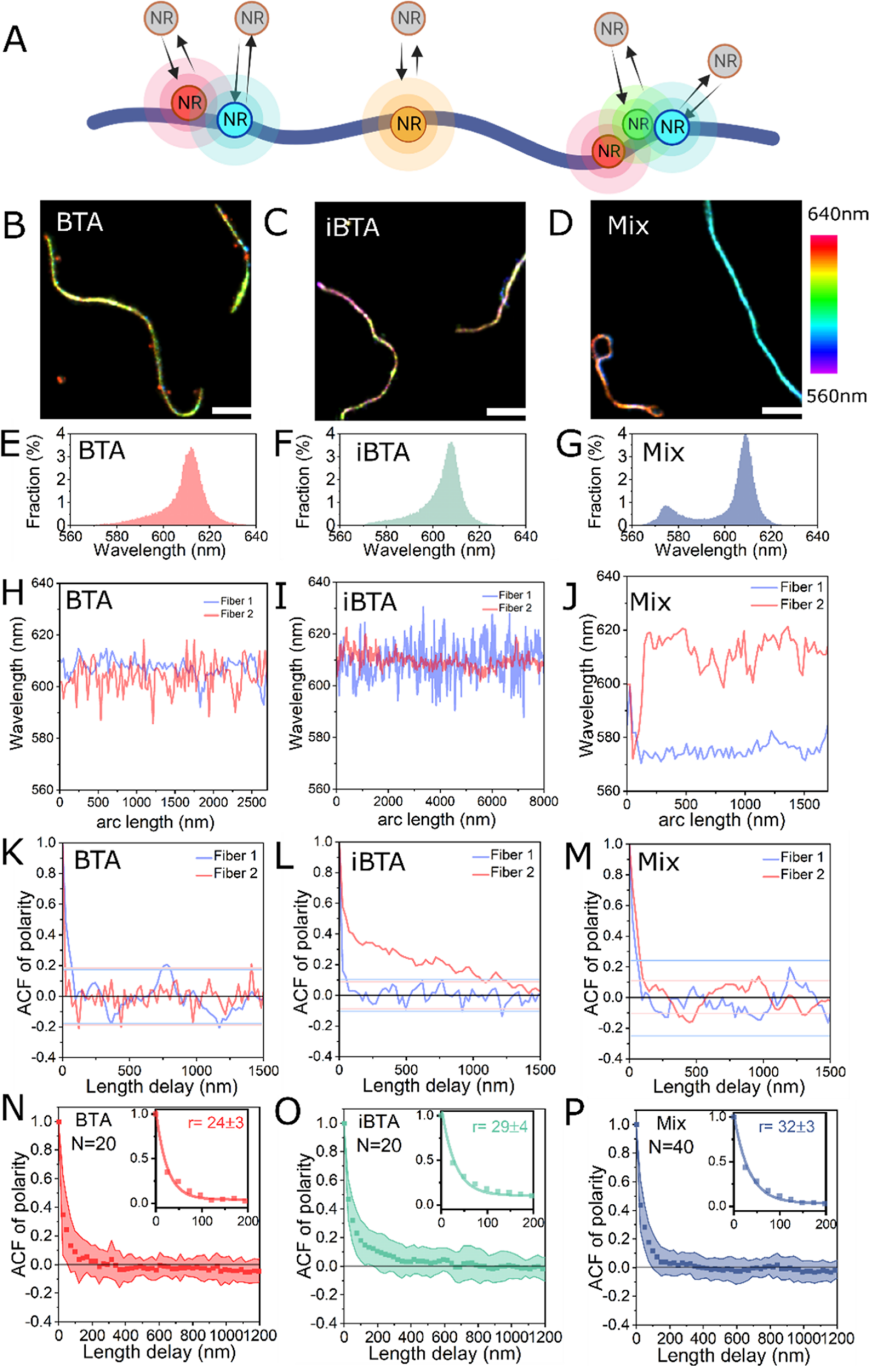
Heterogeneity analysis of polarity along the polymer fibers, between fibers, and between polymer categories. (A) Schematic of NR dyes probing the polarity along the polymer fiber by color shift. (B–D) Polarity maps of BTA, iBTA, and BTA–iBTA copolymers probed by Nile Red emission shift in wavelength. The scale bar is 1 μm; frame 32,000. (E–G) Histogram of the emission wavelength of events captured with BTA, iBTA, and BTA–iBTA copolymers. (H–J) Typical polarity profiles of singe copolymer fibers with different emission wavelengths. (K–M) Autocorrelation functions (ACFs) of the polarity profiles of polymers shown in panels (H–J). Confidence bounds are depicted in colored lines. (N–P) Mean autocorrelation function of the polarity as a function of the space-lag for BTA, iBTA, and BTA–iBTA copolymers. Inset figures show a zoomed-in autocorrelation functions with a single-exponential decay fitting. The characteristic decay lengths for BTA, iBTA, and BTA–iBTA copolymers are 24 ± 3, 29 ± 4, and 32 ± 3 nm, respectively.

Second, we examine the polarity profile of the polymer fibers by mapping the NR emission peak (Figure 4A). Typical polarity maps of BTA, iBTA, and BTA–iBTA copolymer are shown in Figure 4B–D. Examples of other polarity maps of polymers can be found in Figures S4–S6. The false color in wavelength reflects the local polarity of the polymer as the emission of NR dyes blue-shifts in a nonpolar environment. In other words, shorter wavelength indicates a less polar local environment. In stark contrast to the relatively homogeneous polarity among the BTA and iBTA polymers, BTA–iBTA copolymers show distinct heterogeneity between fibers. This is clearly evidenced in the histogram of polarities in Figure 4E–G. Although major emission peaks are similar among 3 different types of the polymers at 607, 611, and 608 nm for iBTA, BTA, and copolymer, respectively. In the case of copolymers, an additional population at 575 nm appears more pronounced than the counterparts in BTA and iBTA. This agrees with the fluorescence measurements on the ensemble samples with NR dyes (Figure S8).

Looking at the polarity profiles of single polymers (representative profiles in Figure 4H–J), surprisingly we found that the polarities along the same fibers are rather homogeneous with small fluctuations within 20 nm. The homogeneity at the intrafiber level is in stark contrast to the highly fluctuated event density profiles, which implies that the local polarity is rather unaffected in the disordered regions. The heterogeneity is only observed at the interfiber level, where a small population of copolymer fibers blue-shifts by approximately 40 nm (fibers 1 and 2 in Figure 4J). The small population of BTA–iBTA copolymers with abnormal polarity is plausibly due to the mismatch between the two monomers causing ancillary changes along the fibers.

To get a quantitative overview, we performed ACF analysis of polarity on single fibers (Figure 4K–M) and averaged ACF of polarity on 20 BTA fibers, 20 iBTA fibers, and 40 BTA–iBTA copolymers (Figure 4N–P). As we can see, most ACF curves decay rather quickly, showing a relatively short-distance correlation. The averaged ACF curves can be well fitted with a single-exponential decay function, yielding characteristic lengths of 24 ± 3, 29 ± 4, and 32 ± 3 nm for BTA, iBTA, and BTA–iBTA copolymers, respectively. Notably, these characteristic lengths are similar to the uncertainty of the localization (∼25 nm shown in Figure S3). Therefore, such characteristic lengths reflect rather the limit of spatial precision of the method. This indicates that the polarity along the polymers is random with no detectable structural features longer than 25 nm. A high homogeneity of the polymer polarity is observed at the intrafiber level.

At the interfiber level, we noticed that, among iBTA fibers, a couple of ACFs of polarity have significantly longer decay lengths in comparison to the rest of the population, e.g., the fiber2 in Figure 4L. As can be seen in the polarity profile of the fiber2 in Figure 4I, there is an overall decreasing trend in polarity along this fiber, although the shift in polarity is small. This is a feature similar to the event density profile of the iBTA polymers as discussed in the previous section. In the following section, we are going to further investigate the cross-correlation between the spatial characteristics and the polarity of the polymer fibers.

### Correlation between Spatial and Polar Properties of Fibers

**Figure 5:**
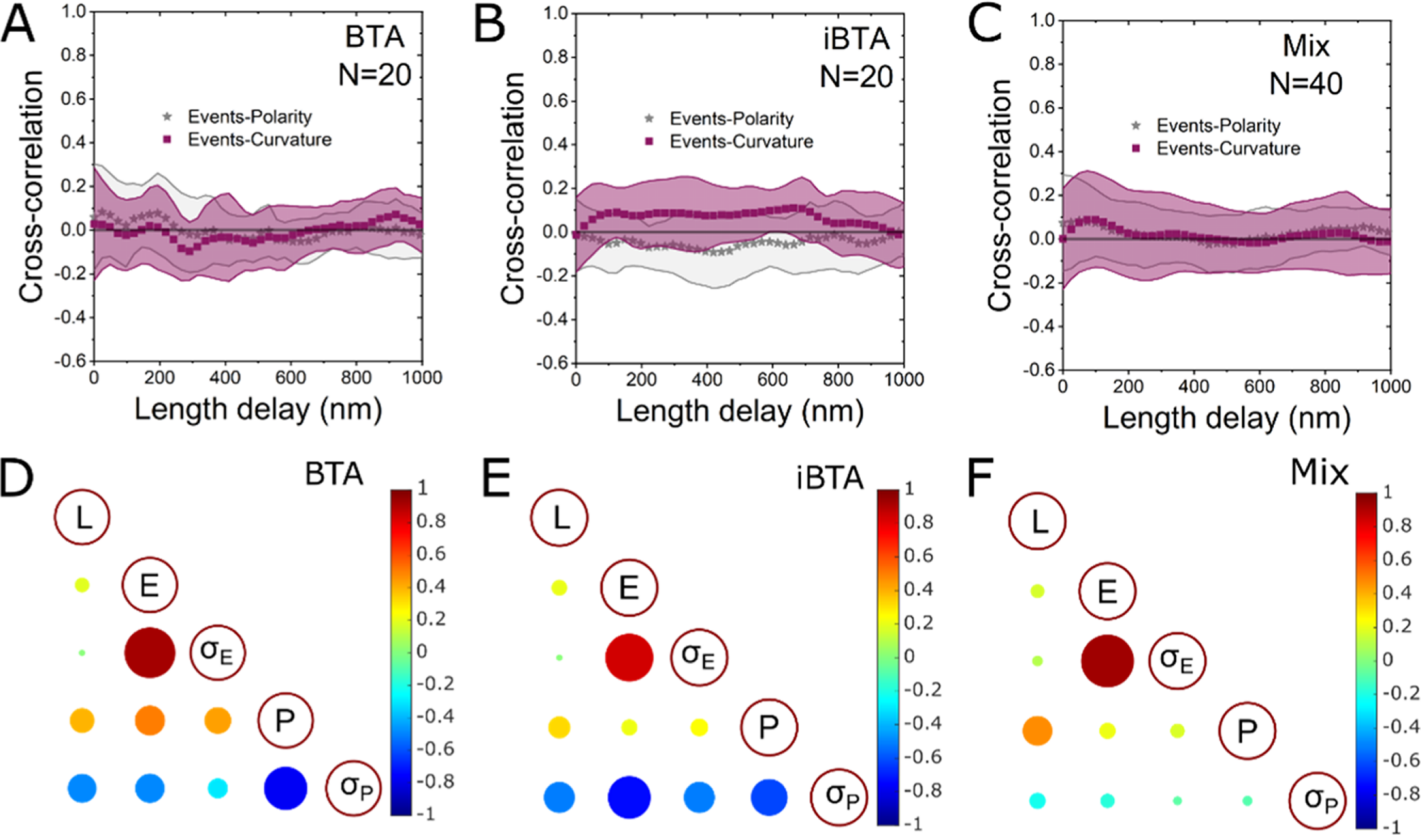
Correlation between spatial and polar properties of polymer fibers. (A–C) Mean cross-correlation between event density and polarity, and event density and curvature along fibers as a function of the distance between each space-lag. Analysis is based on data extracted from 20 BTA fibers (A), 20 iBTA fibers (B), and 40 copolymers (C). (D–F) Correlation matrices analyze correlation at the interfiber level, including arc length (*L*), mean event density per fiber (*E*), standard deviation of event density per fiber (σ_E_), mean polarity per fiber (*P*), and standard deviation of polarity per fiber (σ_p_). The color and the size of the dots are based on values of Pearson’s r, ranging from −1 to 1.

To get a deeper understanding of the polymer property that is probed by the NR binding, a couple of questions need to be answered. Is higher event density associated with the curvature of the polymer and/or the polarity changes? To answer these questions, we investigate the correlation between spatial and polar properties of the polymer fibers. First, we checked the cross-correlation between the spatial and polar properties of the polymers at an intrafiber level. Figure 5A–C depicts the averaged cross-correlations between event density and polarity along the fiber, as well as event density and polymer curvature for all 3 types of polymers. Both cross-correlation values are close to zero, showing negligible cross-correlations on average regardless of the polymer types. However, relatively large heterogeneity between fibers is depicted by the standard deviations of the cross-correlation functions, ranging from −0.3 to 0.3. Cross-correlation between event density and curvature of individual fibers can be found in Figure S10. This suggests that although a weak cross-correlation between spatial and polar properties can be found on some of the polymers, statistically the event density does not tend to correlate with the polymer curvature nor the polarity along the polymer fibers. This means that the local hotspots for NR binding on polymers have a higher accessibility for the NR dyes, while the polarity remains more or less intact.

Second, we explore the correlation between spatial and polar properties of the polymers at an interfiber level through scatter plot matrix analysis. In Figure 5D–F, we paired five characteristics per fiber to investigate the correlations, including arc length (*L*), mean event density per fiber (*E*), standard deviation of event density per fiber (σ_E_), mean polarity per fiber (*P*), and standard deviation of polarity per fiber (σ_P_). Pearson’s correlation coefficient (*r*) is utilized to quantify these correlations with the value ranges from −1 to 1. For all three types of polymers, very strong positive correlations (*r* > 0.8) between the mean event density per fiber (*E*) and the standard deviation of event density per fiber (σ_E_) are observed. It could be indicative of a random process of NR dye binding that obeys the Poisson distribution. On the contrary, strong negative correlations (−0.8 < *r* < −0.6) between polarity per fiber (*P*) and the standard deviation of polarity per fiber (σ_P_) are observed on BTA and iBTA polymers. This implies that, for both BTA and iBTA polymers, fibers with higher polarity on average (more hydrophilic environment) tend to have a more consistent or uniform polarity across fibers, while lower polarity (more hydrophobic environment) on average is associated with greater variability or fluctuation in polarity among fibers. This reflects the heterogeneity of polarity at the interfiber level. For the BTA–iBTA copolymer, the mean polarity per fiber shows a moderate positive correlation with the arc length of the copolymer fibers. In other words, the abnormal population of the copolymers with a large blue shift, i.e., more nonpolar fibers, is only observed in short fibers (Figure S13). Excluding these blue-shift copolymers, the scatter plot matrix of the BTA–iBTA copolymer reveals a resemblance to the patterns observed in BTA and iBTA polymers (Figure S14). Scatter plot matrices with the actual distribution of data for all polymer samples can be found in Figures S11–S14.

For iBTA polymers, a unique strong negative correlation (*r* = −0.74) between the mean event density per fiber (*E*) and the standard deviation of polarity per fiber (σ_P_) is observed (Figure 5E). This correlation suggests that iBTA polymers with higher mean event density are associated with less variability or fluctuation in polarity among fibers. This is also visible in Figure 1C. The correlation coefficients of these pairs are rather low for the BTA polymers and the BTA–iBTA copolymers as *r* = −0.49 and *r* = −0.17, respectively. Further studies are needed to understand the underlying mechanisms for these observed correlations.

## Conclusions

**Figure 6:**
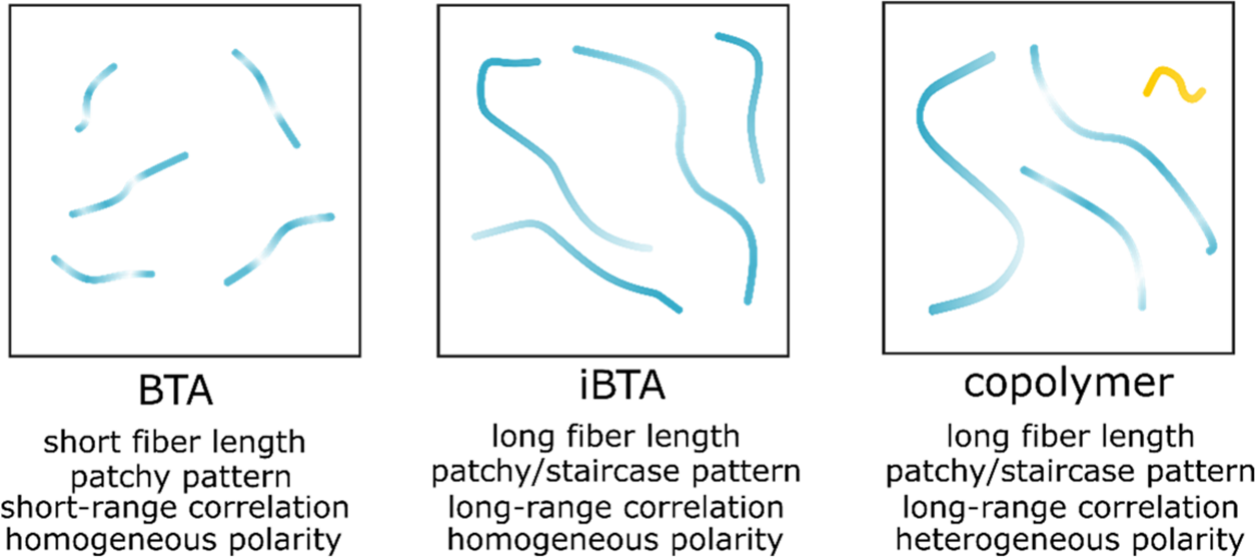
Schematics of the structural and the polar features of BTA, iBTA homopolymers, and BTA–iBTA copolymers.

In conclusion, supramolecular polymers have a complex dynamic structure due to noncovalent interactions between the monomers. A mild disruption of order in the hydrophobic region, intrinsically generated by the monomer design, can lead to an amplification of complex dynamics, as seen here with the inverted amide of BTA/iBTA polymers. The local variations in monomer assembly could be easily distinguished using advanced sPAINT imaging with Nile Red as a polarity-sensitive probe. We summarize our findings of the structural and the polar characteristics of the BTA, iBTA homopolymers, and copolymer in Figure 6. In brief, BTA polymers have shorter arc lengths in comparison to the iBTA polymers and the BTA/iBTA copolymers. Structural variations associated with accessibility of NR dyes are mostly seen in patchy patterns in all types of polymers with subtle differences. In BTA polymers, the patchy patterns have a relatively short-range correlation, whereas iBTA polymers and copolymers have long-range correlations in structure variation. However, the variety in the distribution of disordered domains is significant between individual polymer fibers. In stark contrast, the polarity probed by NR dyes along the polymer chains is homogeneous for all types of polymers. Only heterogeneity is observed at the interfiber level with a small population of less polar (more hydrophobic) fibers in the copolymers.

This study echoes with the recent discovery of the heterogeneity in artificial supramolecular polymers composed of identical monomers and sharing the same shape. Such observations have also been made in biological polymers, such as microtubules.^12^ NR-sPAINT reveals clear heterogeneity in monomer packing at the interfiber level and even at the intrafiber level. Such structural variations are likely to induce differences in functional properties.^48^ Therefore, our work emphasizes the necessity of delving into the nanoscale structure of soft matter using advanced techniques such as NR-sPAINT to fully understand their intricacies.

## Materials and Methods

### Materials

All reagents and chemicals were obtained from commercial sources at the highest purity available and used without further purification unless stated otherwise. All solvents were of AR quality and purchased from Biosolve. 1,2-Dioleoyl-*sn*-glycero-3-phosphocholine (DOPC) and 1,2-dioleoylsn-glycero-3-phosphoethanolamine-*N*-(cap biotinyl), sodium salt (DOPE-biotin) were obtained from Avanti Polar Lipids. A μ-Slide 8 Well plate for total internal reflection fluorescence (TIRF) microscopy was obtained from Ibidi. Water was purified on an EMD Millipore Milli-Q Integral Water Purification System.

### Synthesis

The synthesis of BTA, iBTA, BTABio, BTACy5, and iBTACy3 has previously been reported.^6,37,40^

### Sample Preparation of BTA, iBTA, and Copolymer

For BTA and iBTA homopolymers: stocks (BTA in MeOH and iBTA in ACN) were prepared. A required volume of stock was added to a glass vial, and the solvent was evaporated with a nitrogen flow. To it, MQ water was added to obtain the desired concentration of 50 μM and the samples were subsequently stirred at a temperature of 80 °C for 15 min. The samples were left to equilibrate at room temperature overnight and were further equilibrated in the fridge for 1 week until the measurement.

For fluorescent homopolymers: stocks (BTA in MeOH and iBTA in ACN) were prepared. The required volume of stock was added to a glass vial, and the solvent was evaporated with a nitrogen flow. To it, MQ water was added to obtain the desired concentration of 50 μM and the samples were subsequently stirred at a temperature of 80 °C for 15 min. The hot and hazy samples were vortexed for 15 s before the addition of BTACy5 or iBTACy3 was added from a 1 mM stock solution in MeOH in the desired concentration. The samples were equilibrated at a temperature of 45 °C for 30 min without stirring. The samples were left to equilibrate at room temperature overnight and were further equilibrated in the fridge for 1 week until the measurement.

For copolymers: an equal volume ratio of the two homopolymers as prepared above was added and incubated for 1 h prior to the experiment.

### Sample Preparation of Anchoring BTA, iBTA, and Copolymer with 1% BTABio

For fluorescent anchoring homopolymers: stocks (BTA in MeOH, iBTA in ACN, BTABio in MeOH) were prepared. The required volume of stock was added to a glass vial (final ratio: 1% BTABio and 99% BTA or iBTA), and the solvent was evaporated with a nitrogen flow. To it, MQ water was added to obtain the desired concentration of 50 μM and the samples were subsequently stirred at a temperature of 80 °C for 15 min. The hot and hazy samples were vortexed for 15 s before the addition of BTACy5 or iBTACy3 was added from a 1 mM stock solution in MeOH in the desired concentration. The samples were equilibrated at a temperature of 45 °C for 30 min without stirring. The samples were left to equilibrate at room temperature overnight and were further equilibrated in the fridge for 1 week until the measurement.

For copolymers: an equal volume ratio of the two homopolymers (44.5% BTA + 44.5% iBTA + 1% BTABio) as prepared above was added and incubated for 1 h prior to the experiment.

### Total Internal Reflection Fluorescence (TIRF) Microscopy

TIRF images in Figure 2 were acquired with a Nikon N-STORM microscopy system. The sample was excited using 561/647 nm laser. Fluorescence was collected using a Nikon ×100, 1.4NA oil-immersion objective and passed through a quad-band-pass dichroic filter (97335 Nikon). Images were recorded with an EMCCD camera (ixon3, Andor, pixel size 0.17 μm). The samples were imaged in a μ-Slide 8 Well plate with No. 1.5 coverslip bottom suitable for microscopy. The samples prepared at a 50 μM concentration were diluted to 2.5 μM in 1× PBS prior to imaging.

### Fluorescence Spectroscopy

The investigations in Figure S1a were carried out with the help of a Varian Cary Eclipse fluorescence spectrometer. A Hellma cuvette with an optical path length of 10 mm × 2 mm and a temperature of 20 °C was employed. NR samples were stimulated at 500 nm, and the emission was recorded from 530 to 700 nm using an 800 V PMT detector. Each measurement was averaged over three scans. NR was added to a 50 μM BTA sample from a 0.25 mM stock solution in MeOH to achieve a concentration of 2.5 μM NR. Before the measurements, the samples were allowed to equilibrate at room temperature for 1 h.

### Hydrogen/Deuterium Exchange Followed by Mass Spectrometry (HDX-MS) Measurements

The experiments in Figure S1 were carried out using a XevoTM G2 QTof mass spectrometer (Waters) with a capillary voltage of 2.7 kV, a cone voltage of 80 V, and an extraction cone voltage of 4.0 V. The source temperature was set at 100 °C, the desolvation temperature at 400 °C, the cone gas flow at 10 L/h, and the desolvation gas flow at 500 L/h. BTA samples were diluted with D_2_O (including 0.5 mM sodium acetate to facilitate detection) to a concentration of 50 μM. Aliquots of these 50 μM samples were taken at specific time points and were subjected to electrospray ionization MS. The sample solutions subjected to H/D exchange were introduced into the mass spectrometer using a Harvard syringe pump (11 Plus, Harvard Apparatus) at a flow rate of 50 μL/min. All samples were stored at room temperature during the course of the HDX-MS experiments. Before each measurement, the system was calibrated with a 0.05% H3PO4 solution in 1:1 H2O:ACN. Samples were injected with a syringe pump, and the signal was left to equilibrate for 1 min before the measurement and each measurement was averaged over 1 min to account for instabilities.

### Sample Preparation for NR-sPAINT

Small unilamellar vesicles (SUVs) are first prepared. Separate stock solutions (0.1 mg/mL) of the lipid molecules DOPC and DOPE-biotin were made by dissolving the needed quantity of lipid in chloroform. In a glass vial, the appropriate volume from stocks was combined for the required molar ratio. To obtain a lipid film, chloroform was vaporized from the sample using a nitrogen flow. For 1 h, the vial was vacuum-dried at room temperature. After drying, the lipid film was resuspended in MQ water at a concentration of 1 mg/mL. The solution was then extruded at least 11 times over a 0.1 m membrane filter. The SUVs were kept in the refrigerator at 4 °C for a week. DOPC/DOPE-biotin molar ratio = 99.9:0.1. Ibidi well plates were cleaned by immersing and sonicating for 10 min in (i) 2% w/v sodium dodecyl sulfate (SDS) solution, (ii) 70% v/v ethanol solution, and (iii) MQ water (after thorough rinsing with MQ water). The well plates were then dried under a nitrogen stream and activated by UV/ozone treatment for 10 min.

The cleaned and activated well plates are filled with 200 μL of 2 M NaOH solution and incubated at room temperature for at least 1 h to make the surface hydrophilic for vesicle rupture. The wells are then rinsed thoroughly with MQ water at least 3 times to remove excess NaOH present in wells.

The SUV solution of 1 mg/mL in MQ water is diluted to 0.1 mg/mL with 10 mM PBS just prior to the supported lipid bilayer (SLB) preparation. SUV solution (200 μL) is added to the wells and incubated at room temperature for 30 min for the vesicle fusion-driven SLB formation. The wells are then carefully washed with 10 mM PBS three times to eliminate any SUVs or lipids from the wells. Next, the SLBs are injected with 10 μL of 1 μM streptavidin (SAv) in 10 mM PBS and incubated for 5 min. Since the biotin–streptavidin interaction is very strong, longer incubation times are not necessary. The wells are then carefully washed with 10 mM PBS three times to remove excess SAv. The SAv-appended SLB is kept under 10 mM PBS with no bubbles for stability.

Next, the 50 μM fiber sample of 1%BTABio-functionalized BTA, iBTA, or BTA–iBTA copolymer in MQ (mentioned above) is diluted to 5 μM in 10 mM PBS and added to the prepared SLBs. The well plates are incubated for 5 min and then carefully washed with 10 mM PBS three times to remove excess fibers. Finally, 2.5 nM of Nile Red solution in 10 mM PBS is added to the well plate prior to imaging.

### Nile Red Spectrally-Resolved Point Accumulation for Imaging in Nanoscale Topography (NR-sPAINT)

NR-sPAINT fluorescence imaging was performed using the previously described inverted ide-field TIRF optical microscope (Nikon Ti) with a 532 nm laser source (FP1280764, Coherent OBIS). Excitation was directed off a dichroic mirror (ZET 532/10×) through an oil-immersion objective lens (Nikon Apo TIRF 100× Oil DIC N2) with a numerical aperture of 1.55 and a magnification of 100×. The sample was illuminated in total internal reflection fluorescence (TIRF), with a 532 nm continuous fiber-coupled wavelength source (FP1280764, Coherent OBIS) at 40 mW. The fluorescence emission was collected through the same objective and passed through a long-pass filter (ET542LP, Chroma Technology) and a notch filter (NF533–17, Thorlabs) before being expanded by a 1.5× relay lens. Finally, the emission was passed through a mechanical slit (VA100C, Thorlabs) and a transmission diffraction grating (70 grooves/mm, 25 mm × 25 mm-46–068, Edmund), which was integrated ∼2 cm before an EMCCD camera (Andor DU-888 X-9414). The grating split the emission signal 41% into the zeroth order and 32% into the first order. Camera settings: electron multiplication gain of 250, exposure time of 40 ms, and total frames per image 32,000 frames.

### Single-Fiber Quantification

Spatial domain: the spectrally super-resolved reconstructions were generated using common SMLM fitting algorithms (ThunderSTORM, ImageJ). After proper density cleaning of the background and subsequent fiber identification and clustering, a complete background-free file containing all single-molecule coordinates **{X(nm)**, **Y(nm)**, **T(frames)}** was obtained. The detailed procedure and analysis routine consists of several dependent scripts, which can be run in MATLAB and are reported in our previous work.^46^

Spectral domain: after obtaining the corresponding clean file from above, the stripes in the spectral domain, which correspond to a single event in the spatial domain, were first analyzed with a common sSMLM spectral fitting algorithm (RainbowSTORM, ImageJ) in combination with a calibration curve to relate the spatial-to-spectra distance (pixels) to actual line widths (intensity vs nanometer) (ref). Lastly, all calibrated line widths were fitted with a Gaussian peakfit function to extract the corresponding single-molecule wavelengths. The complete retrieved information from single molecules was further imported to OriginLAB for subsequent analysis.

## Supporting Information Available

HDX-MS data analysis; example of raw images of 0th- and 1st-order diffraction of NR binding events; spatial and spectral uncertainty of NR-sPAINT; examples of event density and polarity maps of BTA, iBTA, and copolymers; comparison of arc length, number of events per fiber, and event density between BTA, iBTA, and copolymers; ensemble fluorescence spectra of NR in BTA, iBTA, copolymer, and SUV; the correlation matrices of copolymer excluding a 580 nm population; and scatter plot matrices of BTA, iBTA, and copolymer (PDF)

## References

[ref1] de Greef T. F. A.; Meijer E. W. Supramolecular Polymers. Nature 2008, 453 (7192), 171–173. 10.1038/453171a. 18464733

[ref2] Rieth S.; Baddeley C.; Badjić J. D. Prospects in Controlling Morphology, Dynamics and Responsiveness of Supramolecular Polymers. Soft Matter 2007, 3 (2), 137–154. 10.1039/B615009H. 32680258

[ref3] Aida T.; Meijer E. W.; Stupp S. I. Functional Supramolecular Polymers. Science 2012, 335 (6070), 813–817. 10.1126/science.1205962. 22344437 PMC3291483

[ref4] Kulkarni C.; Meijer E. W.; Palmans A. R. A. Cooperativity Scale: A Structure–Mechanism Correlation in the Self-Assembly of Benzene-1,3,5-Tricarboxamides. Acc. Chem. Res. 2017, 50 (8), 1928–1936. 10.1021/acs.accounts.7b00176. 28692276 PMC5559720

[ref5] Knowles T. P. J.; Waudby C. A.; Devlin G. L.; Cohen S. I. A.; Aguzzi A.; Vendruscolo M.; Terentjev E. M.; Welland M. E.; Dobson C. M. An Analytical Solution to the Kinetics of Breakable Filament Assembly. Science 2009, 326 (5959), 1533–1537. 10.1126/science.1178250. 20007899

[ref6] Albertazzi L.; van der Zwaag D.; Leenders C. M. A.; Fitzner R.; van der Hofstad R. W.; Meijer E. W. Probing Exchange Pathways in One-Dimensional Aggregates with Super-Resolution Microscopy. Science 2014, 344 (6183), 491–495. 10.1126/science.1250945. 24786073

[ref7] Bochicchio D.; Salvalaglio M.; Pavan G. M. Into the Dynamics of a Supramolecular Polymer at Submolecular Resolution. Nat. Commun. 2017, 8 (1), 147 10.1038/s41467-017-00189-0. 28747661 PMC5529520

[ref8] Gasparotto P.; Bochicchio D.; Ceriotti M.; Pavan G. M. Identifying and Tracking Defects in Dynamic Supramolecular Polymers. J. Phys. Chem. B 2020, 124 (3), 589–599. 10.1021/acs.jpcb.9b11015. 31888337

[ref9] Jonkheijm P.; van der Schoot P.; Schenning A. P. H. J.; Meijer E. W. Probing the Solvent-Assisted Nucleation Pathway in Chemical Self-Assembly Pascal. Science 2006, 313, 80–83. 10.1126/science.1127884. 16825566

[ref10] Green M. M.; Reidy M. P.; Johnson R. D.; Darling G.; O’Leary D. J.; Willson G. Macromolecular Stereochemistry: The out-of-Proportion Influence of Optically Active Comonomers on the Conformational Characteristics of Polyisocyanates. The Sergeants and Soldiers Experiment. J. Am. Chem. Soc. 1989, 111 (16), 6452–6454. 10.1021/ja00198a084.

[ref11] Palmans A. R. A.; Meijer E. W. Amplification of Chirality in Dynamic Supramolecular Aggregates. Angew. Chem., Int. Ed. 2007, 46 (47), 8948–8968. 10.1002/anie.200701285. 17935098

[ref12] Motta M. R.; Biswas S.; Schaedel L. Beyond Uniformity: Exploring the Heterogeneous and Dynamic Nature of the Microtubule Lattice. Eur. J. Cell Biol. 2023, 102 (4), 151370 10.1016/j.ejcb.2023.151370. 37922811

[ref13] Wang L.; Shi X.; Wu Y.; Zhang J.; Zhu Y.; Wang J. A Multifunctional Supramolecular Hydrogel: Preparation, Properties and Molecular Assembly. Soft Matter 2018, 14 (4), 566–573. 10.1039/C7SM02358H. 29334109

[ref14] da Silva L. C. E.; Borges A. C.; de Oliveira M. G.; de Farias M. A. Visualization of Supramolecular Structure of Pluronic F127 Micellar Hydrogels Using Cryo-TEM. MethodsX 2020, 7, 101084 10.1016/j.mex.2020.101084. 33102155 PMC7578745

[ref15] Fukui T.; Uchihashi T.; Sasaki N.; Watanabe H.; Takeuchi M.; Sugiyasu K. Direct Observation and Manipulation of Supramolecular Polymerization by High-Speed Atomic Force Microscopy. Angew. Chem., Int. Ed. 2018, 57 (47), 15465–15470. 10.1002/anie.201809165. 30270474

[ref16] Vandaele J.; Louis B.; Liu K.; Camacho R.; Kouwer P. H. J.; Rocha S. Structural Characterization of Fibrous Synthetic Hydrogels Using Fluorescence Microscopy. Soft Matter 2020, 16 (17), 4210–4219. 10.1039/C9SM01828J. 32292943

[ref17] Beuwer M. A.; Knopper M. F.; Albertazzi L.; van der Zwaag D.; Ellenbroek W. G.; Meijer E. W.; Prins M. W. J.; Zijlstra P. Mechanical Properties of Single Supramolecular Polymers from Correlative AFM and Fluorescence Microscopy. Polym. Chem. 2016, 7 (47), 7260–7268. 10.1039/C6PY01656A.

[ref18] Da Silva R. M. P.; Van Der Zwaag D.; Albertazzi L.; Lee S. S.; Meijer E. W.; Stupp S. I. Super-Resolution Microscopy Reveals Structural Diversity in Molecular Exchange among Peptide Amphiphile Nanofibres. Nat. Commun. 2016, 7, 11561 10.1038/ncomms11561. 27194204 PMC4874009

[ref19] Dhiman S.; Andrian T.; Gonzalez B. S.; Tholen M. M. E.; Wang Y.; Albertazzi L. Can Super-Resolution Microscopy Become a Standard Characterization Technique for Materials Chemistry?. Chem. Sci. 2022, 13 (8), 2152–2166. 10.1039/D1SC05506B. 35310478 PMC8864713

[ref20] Sarkar A.; Sasmal R.; Empereur-mot C.; Bochicchio D.; Kompella S. V. K.; Sharma K.; Dhiman S.; Sundaram B.; Agasti S. S.; Pavan G. M.; George S. J. Self-Sorted, Random, and Block Supramolecular Copolymers via Sequence Controlled, Multicomponent Self-Assembly. J. Am. Chem. Soc. 2020, 142 (16), 7606–7617. 10.1021/jacs.0c01822. 32233467

[ref21] Hanssen J. N. S.; Dhiman S. Impact of Subtle Intermolecular Interactions on the Structure and Dynamics of Multicomponent Supramolecular Polymers. Chem. Commun. 2023, 59 (90), 13466–13469. 10.1039/D3CC04567F. 37877229

[ref22] Sharonov A.; Hochstrasser R. M. Wide-Field Subdiffraction Imaging by Accumulated Binding of Diffusing Probes. Proc. Natl. Acad. Sci. U.S.A. 2006, 103 (50), 18911–18916. 10.1073/pnas.0609643104. 17142314 PMC1748151

[ref23] Jungmann R.; Steinhauer C.; Scheible M.; Kuzyk A.; Tinnefeld P.; Simmel F. C. Single-Molecule Kinetics and Super-Resolution Microscopy by Fluorescence Imaging of Transient Binding on DNA Origami. Nano Lett. 2010, 10 (11), 4756–4761. 10.1021/nl103427w. 20957983

[ref24] Delcanale P.; Miret-Ontiveros B.; Arista-Romero M.; Pujals S.; Albertazzi L. Nanoscale Mapping Functional Sites on Nanoparticles by Points Accumulation for Imaging in Nanoscale Topography (PAINT). ACS Nano 2018, 12 (8), 7629–7637. 10.1021/acsnano.7b09063. 30048592

[ref25] Eklund A. S.; Ganji M.; Gavins G.; Seitz O.; Jungmann R. Peptide-PAINT Super-Resolution Imaging Using Transient Coiled Coil Interactions. Nano Lett. 2020, 20 (9), 6732–6737. 10.1021/acs.nanolett.0c02620. 32787168 PMC7496730

[ref26] Riera R.; Hogervorst T. P.; Doelman W.; Ni Y.; Pujals S.; Bolli E.; Codée J. D. C.; van Kasteren S. I.; Albertazzi L. Single-Molecule Imaging of Glycan–Lectin Interactions on Cells with Glyco-PAINT. Nat. Chem. Biol. 2021, 17 (12), 1281–1288. 10.1038/s41589-021-00896-2. 34764473

[ref27] Filius M.; Cui T. J.; Ananth A. N.; Docter M. W.; Hegge J. W.; van der Oost J.; Joo C. High-Speed Super-Resolution Imaging Using Protein-Assisted DNA-PAINT. Nano Lett. 2020, 20 (4), 2264–2270. 10.1021/acs.nanolett.9b04277. 32168456 PMC7146856

[ref28] Zhang M.; Lihter M.; Chen T. H.; Macha M.; Rayabharam A.; Banjac K.; Zhao Y.; Wang Z.; Zhang J.; Comtet J.; Aluru N. R.; Lingenfelder M.; Kis A.; Radenovic A. Super-Resolved Optical Mapping of Reactive Sulfur-Vacancies in Two-Dimensional Transition Metal Dichalcogenides. ACS Nano 2021, 15 (4), 7168–7178. 10.1021/acsnano.1c00373. 33829760

[ref29] Archontakis E.; Woythe L.; van Hoof B.; Albertazzi L. Mapping the Relationship between Total and Functional Antibodies Conjugated to Nanoparticles with Spectrally-Resolved Direct Stochastic Optical Reconstruction Microscopy (SR-DSTORM). Nanoscale Adv. 2022, 4 (20), 4402–4409. 10.1039/D2NA00435F. 36321150 PMC9552925

[ref30] Martens K. J. A.; Gobes M.; Archontakis E.; Brillas R. R.; Zijlstra N.; Albertazzi L.; Hohlbein J. Enabling Spectrally Resolved Single-Molecule Localization Microscopy at High Emitter Densities. Nano Lett. 2022, 22 (21), 8618–8625. 10.1021/acs.nanolett.2c03140. 36269936 PMC9650776

[ref31] Schoenmakers S. M. C. Supramolecular Polymers under the Magnifying Glass: On the Interplay between Structure, Dynamics and Function; Eindhoven University of Technology: Eindhoven, 2021.

[ref32] Bakker M. H.; Lee C. C.; Meijer E. W.; Dankers P. Y. W.; Albertazzi L. Multicomponent Supramolecular Polymers as a Modular Platform for Intracellular Delivery. ACS Nano 2016, 10 (2), 1845–1852. 10.1021/acsnano.5b05383. 26811943

[ref33] Greenspan P.; Fowler S. D. Spectrofluorometric Studies of the Lipid Probe, Nile Red. J. Lipid Res. 1985, 26 (7), 781–789. 10.1016/S0022-2275(20)34307-8. 4031658

[ref34] Kuo C.; Hochstrasser R. M. Super-Resolution Microscopy of Lipid Bilayer Phases. J. Am. Chem. Soc. 2011, 133 (13), 4664–4667. 10.1021/ja1099193. 21405121 PMC3069148

[ref35] Bongiovanni M. N.; Godet J.; Horrocks M. H.; Tosatto L.; Carr A. R.; Wirthensohn D. C.; Ranasinghe R. T.; Lee J.-E.; Ponjavic A.; Fritz J. V.; Dobson C. M.; Klenerman D.; Lee S. F. Multi-Dimensional Super-Resolution Imaging Enables Surface Hydrophobicity Mapping. Nat. Commun. 2016, 7 (1), 13544 10.1038/ncomms13544. 27929085 PMC5155161

[ref36] Cantekin S.; de Greef T. F. A.; Palmans A. R. A. Benzene-1,3,5-Tricarboxamide: A Versatile Ordering Moiety for Supramolecular Chemistry. Chem. Soc. Rev. 2012, 41 (18), 6125–6137. 10.1039/c2cs35156k. 22773107

[ref37] Leenders C. M. A.; Baker M. B.; Pijpers I. A. B.; Lafleur R. P. M.; Albertazzi L.; Palmans A. R. A.; Meijer E. W. Supramolecular Polymerisation in Water; Elucidating the Role of Hydrophobic and Hydrogen-Bond Interactions. Soft Matter 2016, 12 (11), 2887–2893. 10.1039/C5SM02843D. 26892482 PMC4849209

[ref38] Morgese G.; de Waal B. F. M.; Varela-Aramburu S.; Palmans A. R. A.; Albertazzi L.; Meijer E. W. Anchoring Supramolecular Polymers to Human Red Blood Cells by Combining Dynamic Covalent and Non-Covalent Chemistries. Angew. Chem., Int. Ed. 2020, 59 (39), 17229–17233. 10.1002/anie.202006381. PMC7540258 32584462

[ref39] Vleugels M. E. J.; Varela-Aramburu S.; de Waal B. F. M.; Schoenmakers S. M. C.; Maestro B.; Palmans A. R. A.; Sanz J. M.; Meijer E. W. Choline-Functionalized Supramolecular Copolymers: Toward Antimicrobial Activity against Streptococcus Pneumoniae. Biomacromolecules 2021, 22 (12), 5363–5373. 10.1021/acs.biomac.1c01293. 34846847 PMC8672346

[ref40] Schoenmakers S. M. C.; van den Bersselaar B. W. L.; Dhiman S.; Su L.; Palmans A. R. A. Facilitating Functionalization of Benzene-1,3,5-Tricarboxamides by Switching Amide Connectivity. Org. Biomol. Chem. 2021, 19 (38), 8281–8294. 10.1039/D1OB01587G. 34518862 PMC8494077

[ref41] Lou X.; Lafleur R. P. M.; Leenders C. M. A.; Schoenmakers S. M. C.; Matsumoto N. M.; Baker M. B.; van Dongen J. L. J.; Palmans A. R. A.; Meijer E. W. Dynamic Diversity of Synthetic Supramolecular Polymers in Water as Revealed by Hydrogen/Deuterium Exchange. Nat. Commun. 2017, 8 (1), 15420 10.1038/ncomms15420. 28504253 PMC5440672

[ref42] Lou X.; Schoenmakers S. M. C.; van Dongen J. L. J.; Garcia-Iglesias M.; Casellas N. M.; Romera M. F.-C.; Sijbesma R. P.; Meijer E. W.; Palmans A. R. A. Elucidating Dynamic Behavior of Synthetic Supramolecular Polymers in Water by Hydrogen/Deuterium Exchange Mass Spectrometry. J. Polym. Sci. 2021, 59 (12), 1151–1161. 10.1002/pol.20210011. PMC8247967 34223179

[ref43] Lafleur R. P. M.; Herziger S.; Schoenmakers S. M. C.; Keizer A. D. A.; Jahzerah J.; Thota B. N. S.; Su L.; Bomans P. H. H.; Sommerdijk N. A. J. M.; Palmans A. R. A.; Haag R.; Friedrich H.; Böttcher C.; Meijer E. W. Supramolecular Double Helices from Small C3-Symmetrical Molecules Aggregated in Water. J. Am. Chem. Soc. 2020, 142 (41), 17644–17652. 10.1021/jacs.0c08179. 32935541 PMC7564094

[ref44] Ovesný M.; Křížek P.; Borkovec J.; Švindrych Z.; Hagen G. M. ThunderSTORM: A Comprehensive ImageJ Plug-in for PALM and STORM Data Analysis and Super-Resolution Imaging. Bioinformatics 2014, 30 (16), 2389–2390. 10.1093/bioinformatics/btu202. 24771516 PMC4207427

[ref45] Davis J. L.; Soetikno B.; Song K.-H.; Zhang Y.; Sun C.; Zhang H. F. RainbowSTORM: An Open-Source ImageJ Plug-in for Spectroscopic Single-Molecule Localization Microscopy (SSMLM) Data Analysis and Image Reconstruction. Bioinformatics 2020, 36 (19), 4972–4974. 10.1093/bioinformatics/btaa635. 32663240 PMC7723329

[ref46] Archontakis E.; Deng L.; Zijlstra P.; Palmans A. R. A.; Albertazzi L. Spectrally PAINTing a Single Chain Polymeric Nanoparticle at Super-Resolution. J. Am. Chem. Soc. 2022, 144 (51), 23698–23707. 10.1021/jacs.2c11940. 36516974 PMC9801428

[ref47] Wegner M.; Dudenko D.; Sebastiani D.; Palmans A. R. A.; de Greef T. F. A.; Graf R.; Spiess H. W. The Impact of the Amide Connectivity on the Assembly and Dynamics of Benzene-1,3,5-Tricarboxamides in the Solid State. Chem. Sci. 2011, 2 (10), 2040–2049. 10.1039/c1sc00280e.

[ref48] Jangizehi A.; Schmid F.; Besenius P.; Kremer K.; Seiffert S. Defects and Defect Engineering in Soft Matter. Soft Matter 2020, 16 (48), 10809–10859. 10.1039/D0SM01371D. 33306078

