# Supplementary material for: Visualizing the Heterogeneity in Homogeneous Supramolecular Polymers
Source: J Am Chem Soc. 2024 Jul 10;146(29):19974–85. doi: 10.1021/jacs.4c03562 (PMC11273342; doi:10.1021/jacs.4c03562)
Supplement: Supplementary file 1 — ja4c03562_si_001.pdf [file ja4c03562_si_001.pdf]

Supplementary Information: Visualizing the Heterogeneity In Homogeneous Supramolecular Polymers

Emmanouil Archontakis[a#], Shikha Dhiman[b#†], Miao Zhang[a#], Marle E.J. Vleugels[b], E.W. Meijer[bc], Anja R.A. Palmans[b], Peter Zijlstra[d*], Lorenzo Albertazzi[a*]

a. Department of Biomedical Engineering, and Institute for Complex Molecular Systems, Eindhoven University of Technology, 5600MBEindhoven, The Netherlands

b. Laboratory of Macromolecular and Organic Chemistry, and Institute for Complex Molecular Systems, Eindhoven University of Technology, P. O. Box 513, 5600 MB Eindhoven, The Netherlands

c. School of Chemistry and RNA Institute The University of New South Wales, Sydney, New South Wales 2052, Australia

d. Department of Applied Physics and Science Education, and Institute for Complex Molecular Systems, Eindhoven University of Technology,5600MB Eindhoven, The Netherlands

HDX-MS data analysis

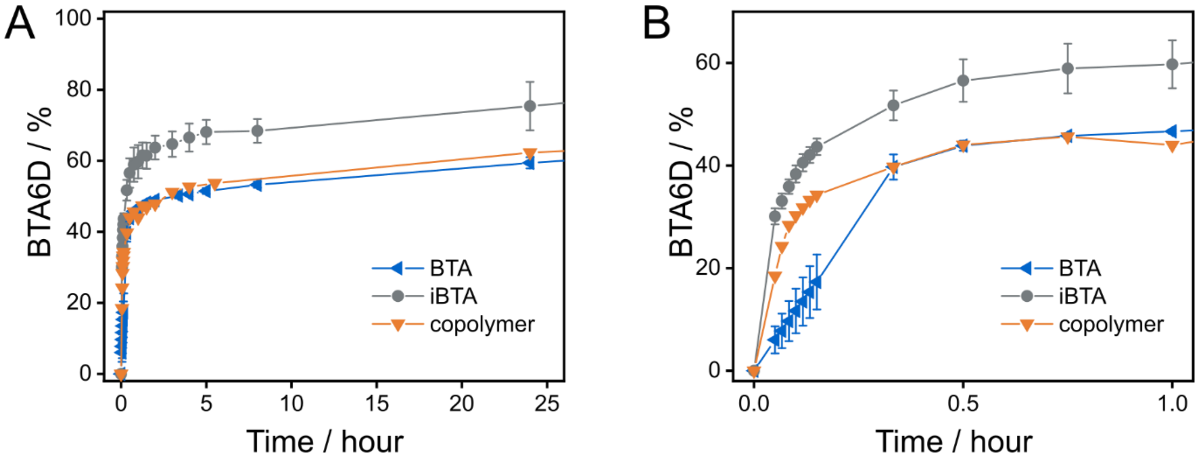

Figure S1. HDX-MS of homopolymers iBTA and BTA and copolymer, after 10 times dilution from MQ water to D_2O. For solubility reasons, 10% of acetonitrile was added to iBTA homopolymers. (initial concentration = 500 µM, after dilution 50 µM). (A) HDX-MS measured over 25 hours. (B) HDX-MS measurement at the initial hour.

All HDX-MS experiments were performed under similar conditions to eliminate influences from the environment, for example temperature. The measurements were performed by diluting the BTA samples 10 times into D_2O. For solubility reasons, 10% of acetonitrile was added to iBTA homopolymers. 0.5 mM sodium acetate was added to D_2O prior to the dilution to facilitate the detection. The H/D exchange was followed over time and the percentage of different deuterated isotopes was calculated. The distribution with two sodium ions was used for the calculations. Isotope distributions were calculated with IsoPro Software. The data for the homopolymers of BTA and iBTA originate from previous reports.[1,2] As BTAs and iBTAs are

constitutional isomers they share the same exact mass and the same set equations can be applied to BTAs and iBTAs. “BTA” can be replaced with “iBTA” in all of the following equations.

The following set of equations was used for the isotope correction of BTA monomers in the copolymer:

$I_{BTA1D\_c} = I_{667.46}$

$I_{BTA2D\_c} = I_{667.96} - 0.80 I_{BTA1D\_c}$

$I_{BTA3D\_c} = I_{668.47} - 0.80 I_{BTA2D\_c} - 0.36 I_{BTA1D\_c}$

$I_{BTA4D\_c} = I_{668.97} - 0.80 I_{BTA3D\_c} - 0.36 I_{BTA2D\_c} - 0.11 I_{BTA1D\_c}$

$I_{BTA5D\_c} = I_{669.47} - 0.80 I_{BTA4D\_c} - 0.36 I_{BTA3D\_c} - 0.11 I_{BTA2D\_c} - 0.03 I_{BTA1D\_c}$

$I_{BTA6D\_c} = I_{669.97} - 0.80 I_{BTA5D\_c} - 0.36 I_{BTA4D\_c} - 0.11 I_{BTA3D\_c} - 0.03 I_{BTA2D\_c} - 0.01 I_{BTA1D\_c}$

With $I_{667.46}$, $I_{667.96}$, $I_{668.47}$, $I_{668.97}$, $I_{669.47}$ and $I_{669.97}$ the intensity at m/z = 667.46, 667.96, 668.47, 668.97, 669.47 and 669.97, respectively.

The following set of equations was used to correct for 10 vol% H2O in the calculations of both components in the copolymer:

$I_{BTA1D} = I_{BTA1D\_c} – 1.02x10^{-4}\ I_{BTA6D\_c} – 3.70x10^{-2}\ I_{BTA3D\_c}$

$I_{BTA2D} = I_{BTA2D\_c} – 2.29x10^{-3}\ I_{BTA6D\_c} – 3.33x10^{-1}\ I_{BTA3D\_c}$

$I_{BTA3D} = I_{BTA3D\_c} – 2.74x10^{-2}\ I_{BTA6D\_c} + (3.70x10^{-2} + 3.33x10^{-1})\ I_{BTA3D\_c}$

$I_{BTA4D} = I_{BTA4D\_c} – 1.85x10^{-1}\ I_{BTA6D\_c}$

$I_{BTA5D} = I_{BTA5D\_c} – 6.67x10^{-1}\ I_{BTA6D\_c}$

$I_{BTA6D} = I_{BTA6D\_c} + (1.02x10^{-4} + 2.29x10^{-3} + 2.74x10^{-2} + 1.85x10^{-1} + 6.67x10^{-1})\ I_{BTA6D\_c}$

The percentage of the deuterated analogues for both BTAs can be calculated with:

$$\%BTAnD = \frac{I_{BTAnD}}{\sum_{k=1}^{6} I_{BTAkD}} \text{ x } 100\%$$

Table S1. Rate of HDX obtained by exponential fit of Figure S1b.

	t_1 (h)	t_2 (h)	k_1 (h^{-1})	k_2 (h^{-1})	R^2
BTA	-	0.273	-	3.7	0.969
iBTA	0.029	0.246	34.5	4.1	0.999
copolymer	0.057	0.256	17.5	3.9	0.992

HDX-MS data is normalized and fit using a biexponential growth function. The analysis reveals that BTA exhibits a mono-exponential growth, while iBTA and copolymer demonstrate a biexponential fit with a two-orders-of-magnitude faster decay component. This observation

implies that the mechanisms governing the exchange of monomers in and out of solution differ for both the monomer and, consequently, the copolymer exhibits a behavior reflective of both individual components.

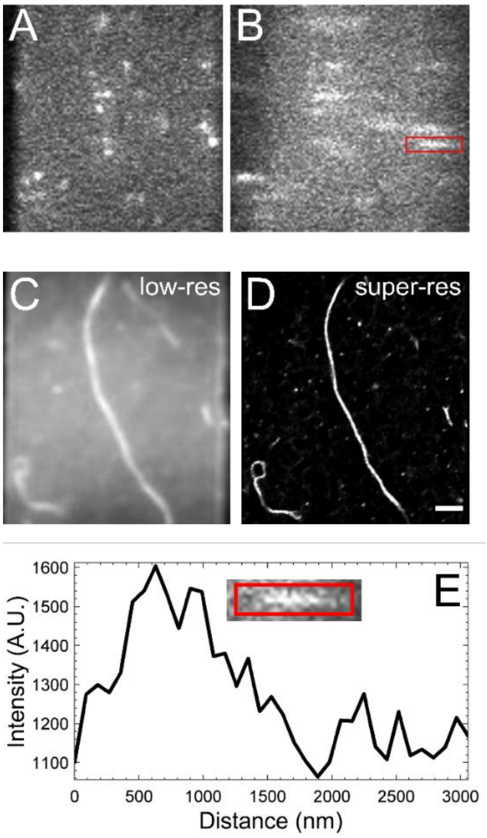

Figure S2. (A) Spatial domain. Each point spread function (PSF) appears as bright puncta which can be fitted to extract the molecule's position. (B) Each dispersed PSF appears as stripe which corresponds to a single event in A). The distance between the points in A) and the stripe in B) corresponds to the wavelength of this specific binding molecule. (C) Diffraction limited fluorescence image of an iBTA sample. (D) Super-resolution image of NR-sPAINT after fitting each individual event in A). (E) A single molecule linewidth taken out from B). Here the distance represents the dispersion of the stripe. Scale bar 500 nm, frames 32000, exposure time 40 ms, laser power 40 mW..

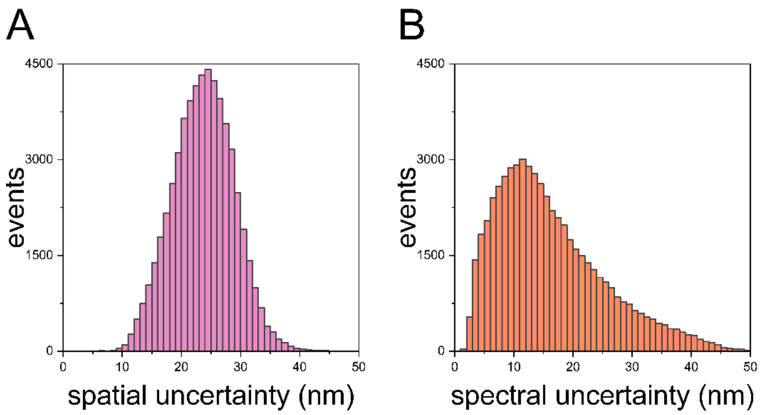

Figure S3. Spatial and spectral uncertainty extracted from a single measurement by counting thousands of molecule positions and spectra. (A) Spatial uncertainty defines the instrument's spatial resolution. (B) Spectral uncertainty defines its spectral resolution.

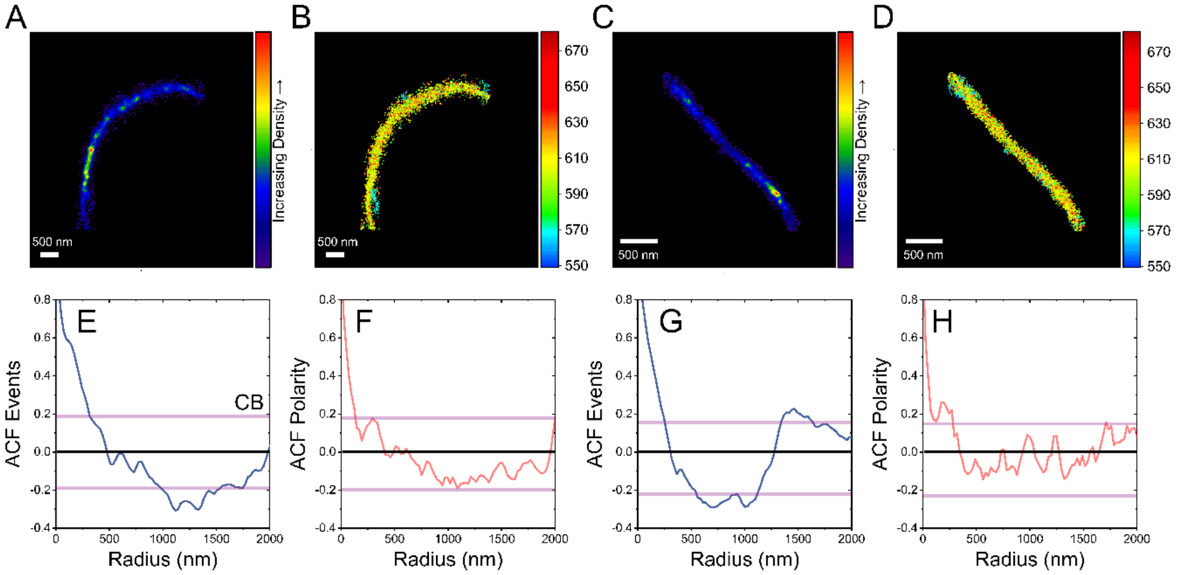

Figure S4. Event density and polarity maps of two BTA fibers. (A) Event density of a relatively low-density sample with a small patchy distribution. (B) Corresponded spectrally resolved map of the fiber in A). (C) Event density of a relatively low-density sample which also exhibits small patchy distribution. (D) Corresponded spectrally resolved map of the fiber in C). Each colored point in B) and D) represents a single wavelength value for the binding event; One molecule at a time, one wavelength at a time. (E-H) Extracted autocorrelation plots (blue for event values, red for polarity values) as a function of the distance between each space-lag (nm). The horizontal lines correspond to the confidence bounds of each measurement. If random, such autocorrelations should be near zero, thus <CB for all space-lag separations. If non-random, then one or more of the autocorrelations will be significant above the CB.

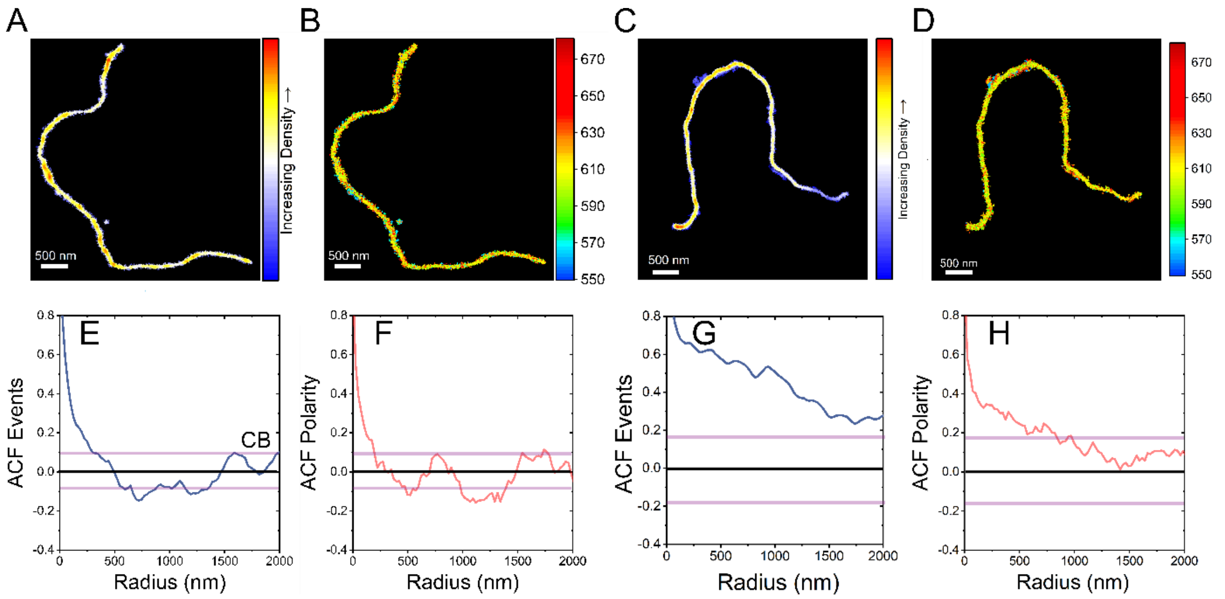

Figure S5. Event density and polarity maps of two different iBTA polymers. (A) Event density of a relatively high-density sample with small clusters of events patchy across the length. (B) Corresponded spectrally resolved map of the fiber in A). (C) Event density of an extreme high-density sample which exhibits a huge patchy distribution. (D) Corresponded spectrally resolved map of the fiber in C). Each colored point in B) and D) represents a single wavelength value for the binding event; One molecule at a time, one wavelength at a time. (E-H) Extracted autocorrelation plots (blue for event values, red for polarity values) as a function of the distance between each space-lag (nm). The horizontal lines correspond to the confidence bounds of each measurement. If random, such autocorrelations should be near zero, thus <CB for all space-lag separations. If non-random, then one or more of the autocorrelations will be significant above the CB.

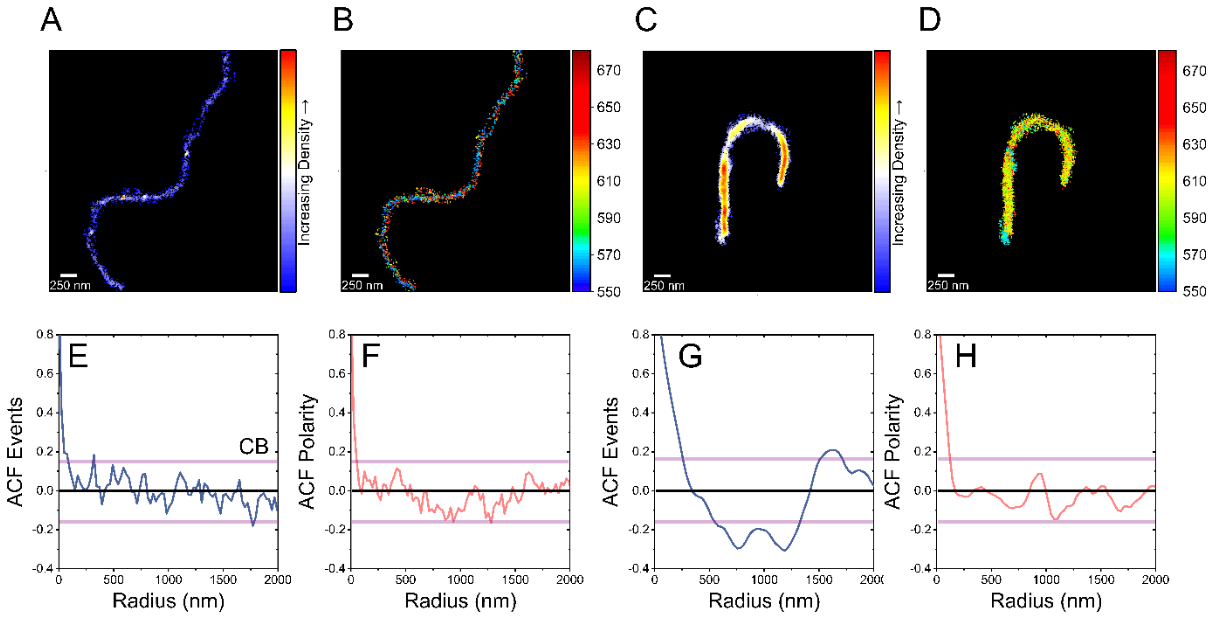

Figure S6. Event density and polarity maps of two different mixed copolymer fibers. (A) Event density of a relatively low-density sample which exhibits a rather homogeneous distribution of events and small clusters of events across its length. (B) Corresponded spectrally resolved map of the fiber in A). (C) Event density of a relatively high-density sample which exhibits a rather patchy distribution of events only at the tail ends. (D) Corresponded spectrally resolved map of the fiber in C). Each colored point in B) and D) represents a single wavelength value for the binding event; One molecule at a time, one wavelength at a time. (E-H) Extracted autocorrelation plots (blue for event values, red for polarity values) as a function of the distance between each space-lag (nm). The horizontal lines correspond to the confidence bounds of each measurement. If random, such autocorrelations should be near zero, thus <CB for all space-lag separations. If non-random, then one or more of the autocorrelations will be significant above the CB.

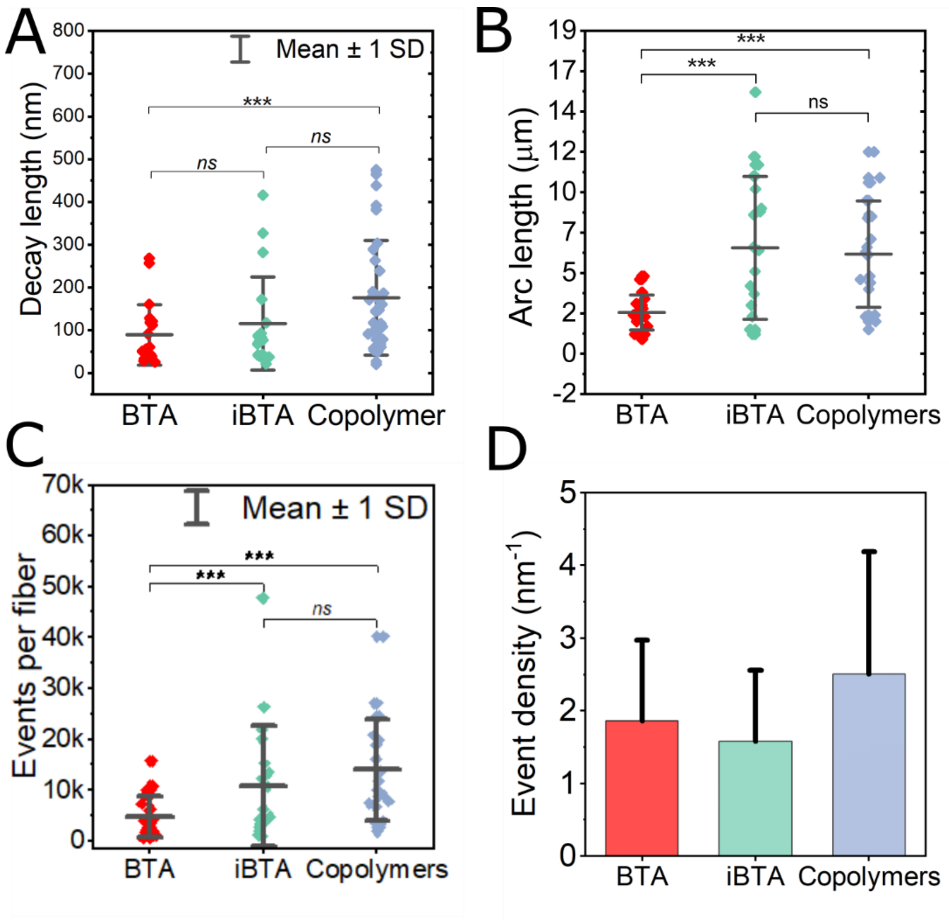

Figure S7. Comparison of decay length, arc length, number of events per fiber, and event density between BTA, iBTA and copolymers:(A) Characteristic decay length per fiber per sample category. The decay lengths were extracted by fitting single exponential decay curve. Each point represents one fiber. Statistically significant difference (P<.0001) was observed between BTA and copolymer samples. (B) Arc length per fiber per sample category. Each point represents one fiber. There is a significant size difference (P< .0003) between BTA and the other two samples (iBTA, copolymers). Notably, there is an interparticle heterogeneity within all samples, which is more intense in iBTA and copolymer ones. (C) Number of events per fiber per sample category. Each point represents one fiber. Significant difference is observed between BTA and the other two samples (iBTA, copolymer), but not between iBTA and copolymers. (D) Averaged event density per sample category.

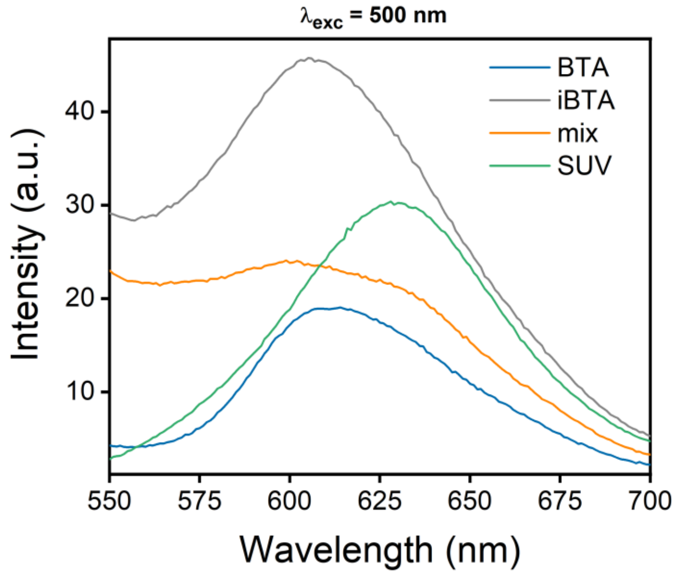

Figure S8. A. Fluorescence emission spectra of NR in homopolymers iBTA (50 µM) and BTA (50 µM), copolymer (50 µM) and small unilamellar vesicles (SUV) (0.1 mg/mL) with 2.5 µM NR.

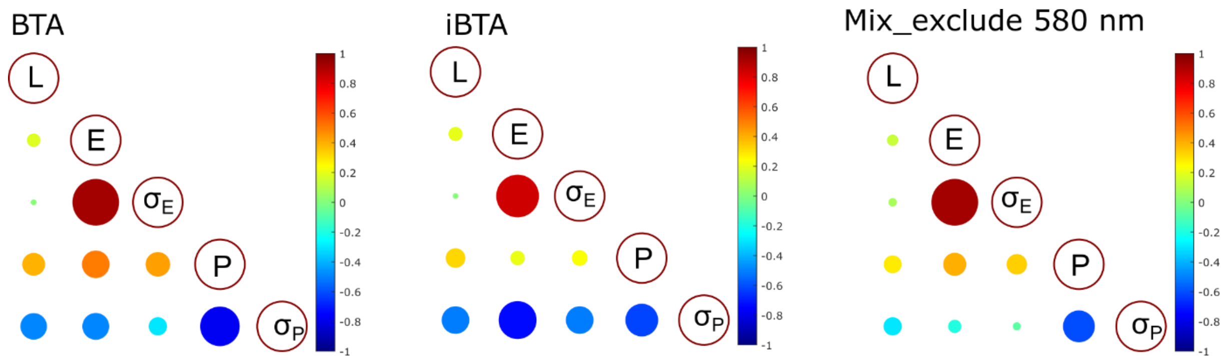

Figure S9. The correlation matrices analyze the inter-fiber correlation for BTA, iBTA and mixed copolymer. Specifically, fibers with polarity below 580 nm are excluded from the copolymer samples in this analysis. The correlation matrices include variables such as arc length (*L*), mean event density per fiber (*E*), standard deviation of event density per fiber (σ_E), mean polarity per fiber (*P*), standard deviation of polarity per fiber (σ_p). The color and the size of the dots are based on values of Pearson's r, ranging from -1 to 1.

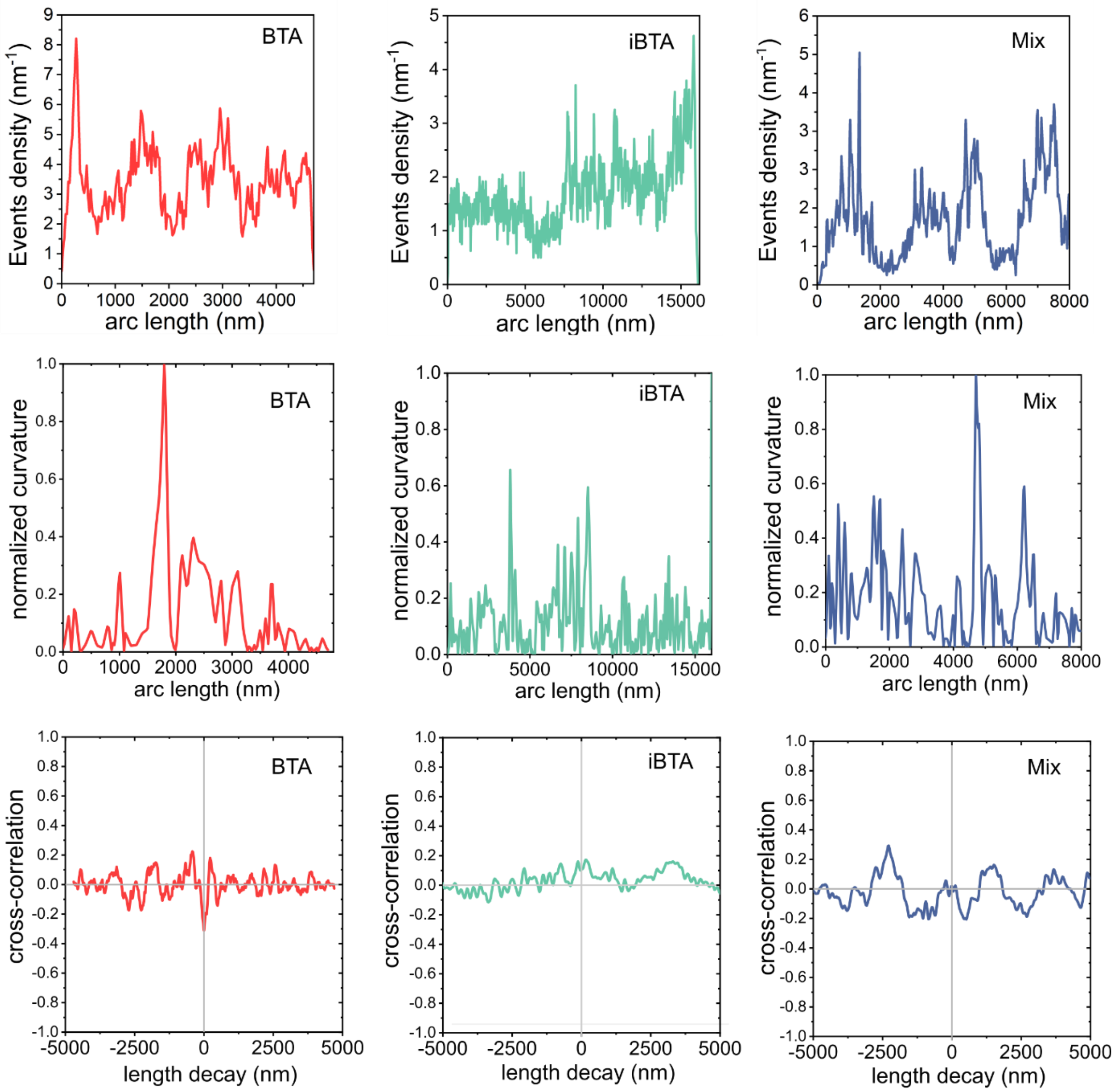

Figure S10. Cross-correlation between event density and curvature of individual fibers. Top panel: event density profiles of BTA, iBTA and copolymer shown in Figure 3B-D. Middle panel: normalized curvature profiles of the same fibers. Bottom panel: cross-correlation curves between event density and curvature of the fibers in the upper panels.

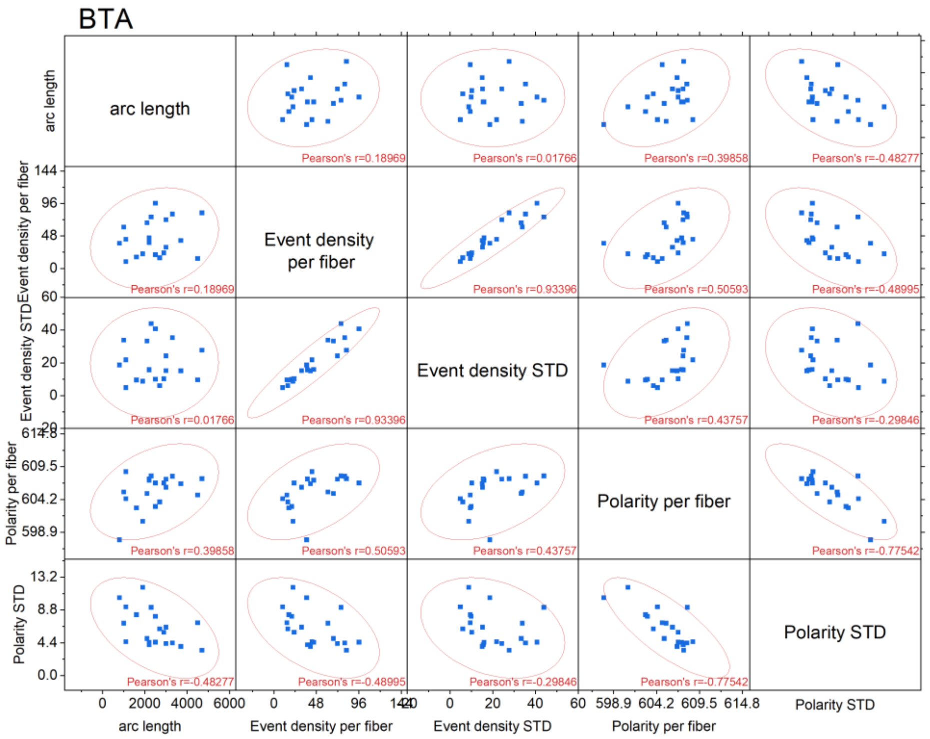

Figure S11. Scatter plot matrix shows the correlation between arc length, mean event density per fiber, standard deviation of event density per fiber, mean polarity per fiber and standard deviation of polarity per fiber, for BTA polymers.

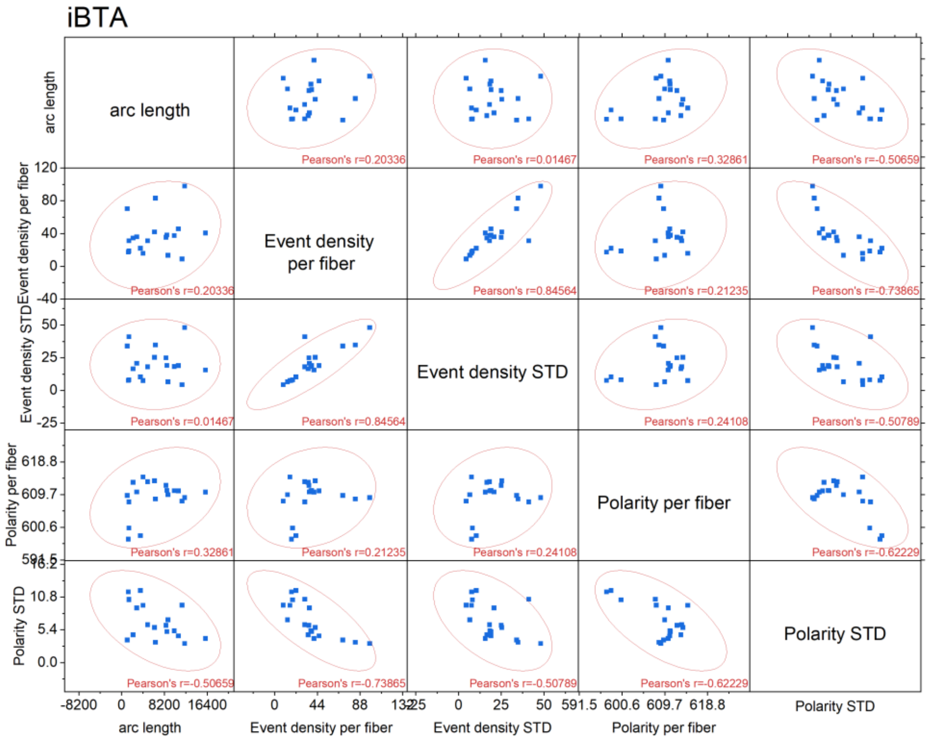

Figure S12. Scatter plot matrix shows the correlation between arc length, mean event density per fiber, standard deviation of event density per fiber, mean polarity per fiber and standard deviation of polarity per fiber, for iBTA polymers.

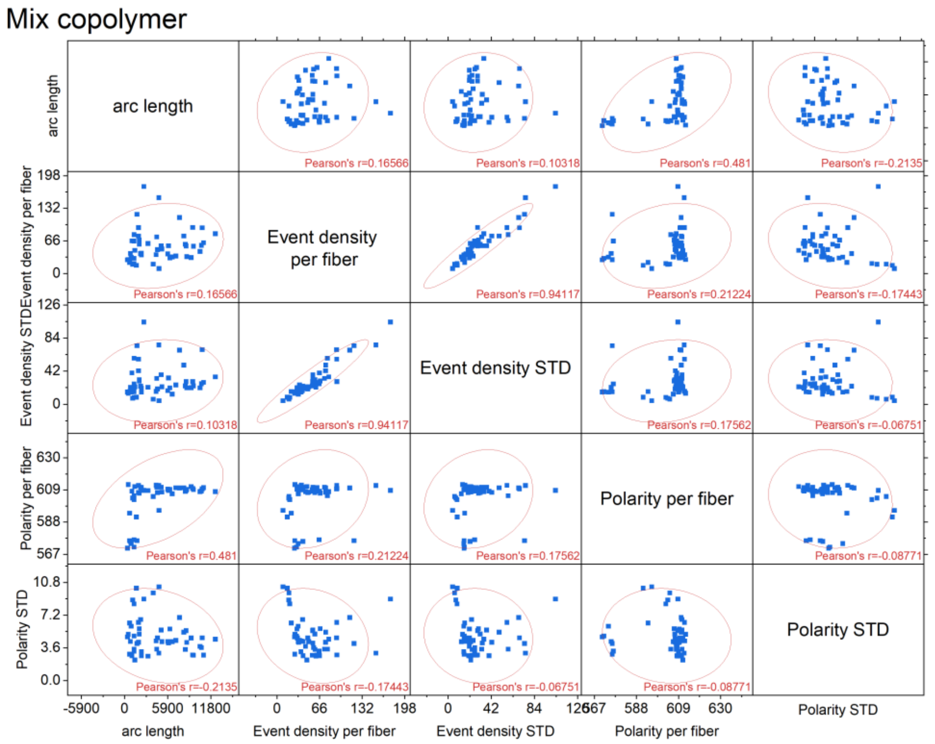

Figure S13. Scatter plot matrix shows the correlation between arc length, mean event density per fiber, standard deviation of event density per fiber, mean polarity per fiber and standard deviation of polarity per fiber, for BTA/iBTA mixed copolymers.

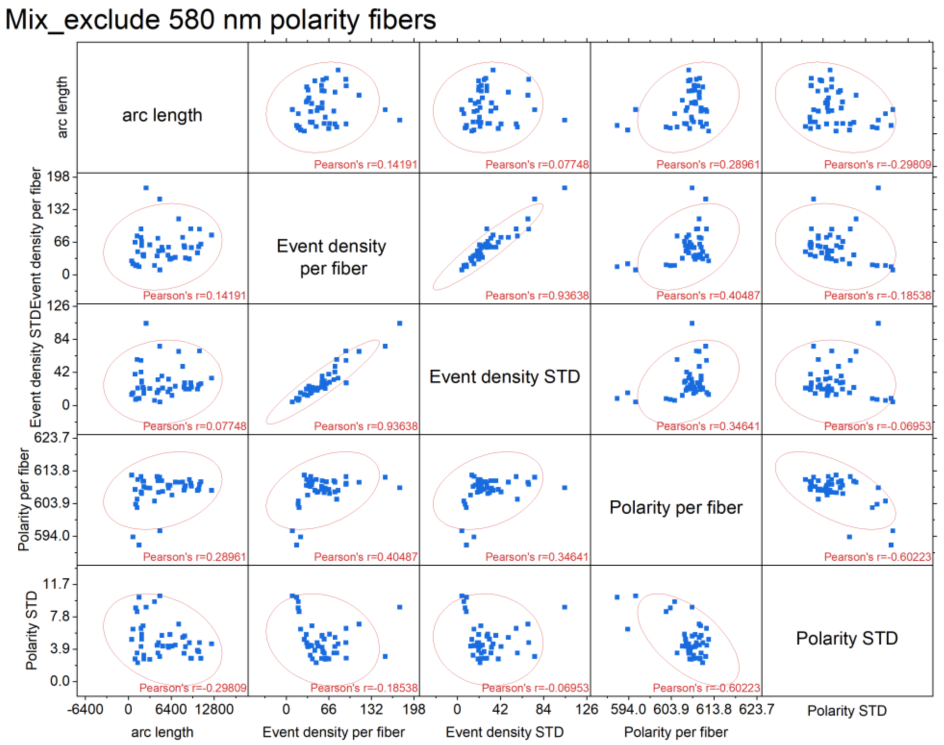

Figure S14. Scatter plot matrix shows the correlation between arc length, mean event density per fiber, standard deviation of event density per fiber, mean polarity per fiber and standard deviation of polarity per fiber, for BTA/iBTA mixed copolymers with exclusion of fibers with polarity below 580 nm.

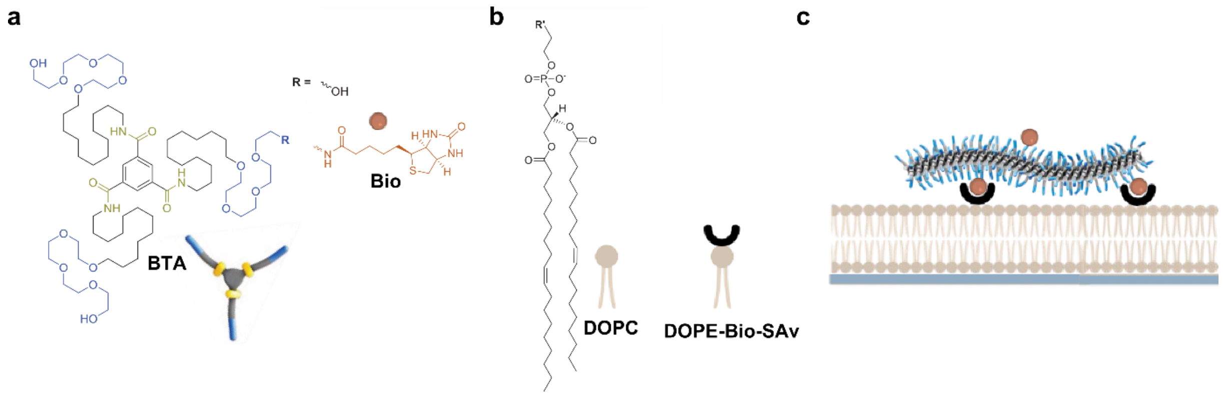

Figure S15. a) chemical structure of BTA and BTABio. b) Chemical structure of DOPC and schematic of DOPE-Bio-Sav. c) Schematic representation of fiber attached to SLB. (figures are partially made by BioRender)

Reference

(1) Lou, X.; Schoenmakers, S. M. C.; van Dongen, J. L. J.; Garcia-Iglesias, M.; Casellas, N. M.; Fernández-Castaño Romera, M.; Sijbesma, R. P.; Meijer, E. W.; Palmans, A. R. A. Elucidating Dynamic Behavior of Synthetic Supramolecular Polymers in Water by Hydrogen/Deuterium Exchange Mass Spectrometry. *J. Polym. Sci.* **2021**, *59* (12), 1151–1161. https://doi.org/10.1002/pol.20210011.

(2) Schoenmakers, S. M. C.; van den Bersselaar, B. W. L.; Dhiman, S.; Su, L.; Palmans, A. R. A. Facilitating Functionalization of Benzene-1,3,5-Tricarboxamides by Switching Amide Connectivity. *Org. Biomol. Chem.* **2021**, *19* (38), 8281–8294. https://doi.org/10.1039/d1ob01587g.
